# Hydroxyurea - Pragmatic Reduction In Mortality and Economic burden (H-PRIME): A 2x2x2 factorial randomised open-label trial investigating practical approaches to the treatment of sickle cell disease at four sites in Eastern Uganda

**DOI:** 10.12688/wellcomeopenres.24096.1

**Published:** 2025-05-16

**Authors:** Peter Olupot-Olupot, Denis Amorut, Paul Ongodia, Charles B. Okalebo, Julian Abeso, Florence Aloroker, Sarah Asio, Amos Odiit, Charles Kiyaga, Rita Muhindo, Sophie Uyoga, George Mochamah, Asad Muyinda, Grace Abongo, Gideon Nyutu, Christabel Mogaka, Kathryn Maitland, Diana Gibb, Ann Sarah Walker, Roisin Connon, Elizabeth George, Russell E Ware, Thomas N. Williams

**Affiliations:** 1 Mbale Campus, Busitema University, Mbale, Eastern Region, Uganda; 2 Mbale Clinical Research Institute, Mbale, Uganda; 3 Uganda Central Public Health Laboratory, Kampala, Uganda; 4 Centre for Geographic Medicine Research Coast, Kilifi, Kilifi County, Kenya; 5 Imperial College London Institute of Global Health Innovation, London, England, UK; 6 Medical Research Council Clinical Trials Unit at University College London, London, England, UK; 7 Cincinnati Children's Hospital Pediatric Hematology and Global Health Center, Cincinnati, USA

**Keywords:** sickle cell disease, hydroxyurea, malaria, bacterial infections, Uganda

## Abstract

**Background:**

Sickle cell disease is the most common and severe genetic disease in humans worldwide. The majority of those affected are born in sub-Saharan Africa, where resources to diagnose and manage them are often limited. Fresh approaches to the pragmatic management of children with sickle cell disease in Africa that improve both morbidity and mortality are urgently needed.

**Methods:**

Hydroxyurea - Pragmatic Reduction In Mortality and Economic burden (H-PRIME) is a 2 × 2 × 2 factorial randomized open-label trial that is investigating three separate interventions. Eighteen hundred children aged 1-10 years attending sickle cell clinics at one of four sites in Eastern Uganda are being randomized to: (1) hydroxyurea administered at a low dose (10 mg/kg/day) versus high dose (25 mg/kg/day), prescribed pragmatically through a weight-band-based approach and with clinically, rather than laboratory-guided monitoring; (2) enhanced malaria prophylaxis with weekly doses of dihydroartemisinin-piperaquine versus monthly doses of sulfadoxine-pyrimethamine (standard of care); and (3) enhanced antibacterial prophylaxis with daily cotrimoxazole throughout life versus twice daily penicillin until the age of 5 years (standard of care). All children will be followed up for 48 months after the date on which the first child was randomized. The primary endpoint for the hydroxyurea randomization will be mortality, for the antimalarial random will be malaria-associated hospital admission, and for the antimicrobial randomization will be all-cause hospital admission. Secondary outcomes will include the incidence of sickle-specific complications, receipt of blood transfusions, hemoglobin and fetal hemoglobin concentrations, and economic costs and benefits of the three interventions.

**Conclusions:**

The first participant was recruited for the trial on January 16, 2024. In total, 1487 of the 1800 target children were recruited by February 17, 2025. H-PRIME will efficiently answer three important pragmatic questions regarding the management of children with sickle cell disease in Africa.

**Registration:**

PACTR number 202401802272880 (09/01/2024).

ISRCTN15724013 (25/02/2020).

## Plain language summary

Sickle cell disease is common in sub-Saharan Africa, where it affects as many as 2% of all births in many countries. Nevertheless, most treatment guidelines within the region are based on research conducted in high-income countries that might not be the most appropriate in Africa, where clinical resources are often more limited and where, for environmental and other reasons, clinical complications may also differ. H-PRIME was designed to address three separate but interlinked questions in a single trial: (1) is a higher dose (25 mg/kg/day) better than a lower dose (10 mg/kg/day) in terms of child survival, and can we deliver a higher dose safely without intensive laboratory monitoring in a low resource setting? (2) can we improve malaria prevention through the use of an alternative approach to prophylaxis: weekly dihydro-artemisinin-piperaquine instead of sulfadoxine-pyrimethamine, the current standard of care? and (3) can we prevent more bacterial infections by using prophylaxis with a broader spectrum antibiotic (daily cotrimoxazole throughout childhood), instead of a narrow spectrum antibiotic (twice daily penicillin until the age of five years)? We address these questions through a light-touch clinical trial conducted in 1800 children 1-10 years old at four clinical centers in Eastern Uganda, who are being simultaneously randomized to all three interventions. The primary endpoint of the high-versus low-dose hydroxyurea questions will be mortality. The primary endpoint of the malaria intervention will be malaria-specific hospital admission, and that for the antibiotic intervention will be all-cause hospital admission (because it is technically and practically difficult to pin down infections to specific bacteria in low-resource settings with limited clinical and laboratory infrastructure). The trial began in January 2024, and 1487 of the 1800 target children were recruited by February 17, 2025.

## Introduction

The term sickle cell disease (SCD) describes a group of conditions in which hemoglobin S (HbS) is the main form of hemoglobin produced. It is characterized by chronic anemia, intermittent crises, and progressive multi-organ deterioration ^1^. HbS is produced as the result of a single base change mutation (A>T: rs334 – the β ^s^ mutation) in codon 6 in *HBB* (HbSS). Unlike normal adult hemoglobin (HbA), abnormal HbS molecules polymerize at low oxygen tension, resulting in abnormal and misshapen “sickled” red blood cells. A number of different forms of SCD can occur, but the most common are caused by the homozygous inheritance of the β ^s^ mutation (HbSS), the co-inheritance of β ^s^ with β−thalassemia (HbS/β-thalassemia) (which together are known as sickle cell anemia; SCA), or the co-inheritance of β ^s^ with other mutations, of which the most common is HbC (HbSC disease) ^1^. Without specific treatment, SCD results in high rates of both disability and early mortality ^1^.

Heterozygous carriers of the β ^s^ mutation (who have sickle cell trait; HbAS) enjoy preferential survival in areas affected by *P. falciparum* malaria ^2^. As a result, the β ^s^ mutation has been selected to high population frequencies in tropical regions of Africa and India, where the majority of children with SCD live ^3^. Most children born with SCD in high-income countries are diagnosed early and treated by specialists. As a result, almost all now survive to adulthood ^4^, largely because of a small number of simple interventions that target common causes of early mortality such as splenic sequestration and bacterial infections ^5– 7^. Nevertheless, wherever they are born, all children with SCD suffer from greater or lesser degrees of chronic ill health, including delayed growth and development, splenic dysfunction, chronic pain (due to tissue hypoxemia), nocturnal hypoxemia, neurocognitive delay, poor school performance, cerebrovascular and parenchymal brain damage, glomerular hyperfiltration, microalbuminuria, and avascular necrosis ^8^. As such, analogous to other childhood medical conditions such as juvenile diabetes, cystic fibrosis or hemophilia, SCD in children and adolescents should be considered a chronic disorder in need of long-term therapy ^8^. Given this, a growing number of treatments are being used to manage patients in high-income countries, including hydroxyurea, chronic transfusion therapy, stem-cell transplantation, gene-therapy or editing, and a large number of potentially promising new drugs ^9, 10^.

The global distribution of the β ^s^ allele means that the majority of children with SCD (>250,000 births annually) are born in sub-Saharan Africa: >75% of all SCD births worldwide ^11^. In marked contrast to high-income countries, new-born screening is rare within this region, the majority of cases are never diagnosed and 50–90% of affected children die before their fifth birthday ^12^. As a result, SCD is a major cause of morbidity throughout most of sub-Saharan Africa, and currently accounts for 5–16% of under-5 mortality overall ^13, 14^. Despite these facts, SCD is widely neglected within the region and many countries have no policies for its routine diagnosis or treatment ^15^. Of the commonly available disease-modifying treatments, all are considered either too complex or too expensive to be realistic options for sub-Saharan Africa within the foreseeable future with only one exception – hydroxyurea – the primary focus of this protocol.

Uganda has the fifth highest burden of SCD globally (10–20,000 annual births) ^7, 16^, wherein the Eastern Region is among the worst affected ^16^. This area typifies SCD-affected populations throughout much of sub-Saharan Africa, where country boundaries are arbitrary in comparison to allele prevalence. Clinical resources within the region are severely constrained, malaria remains hyper-endemic (and recalcitrant to interventions) and under-5 mortality remains high ( ^~^80/1000; national census 2016). Recently, SCD has become a priority issue for the Ugandan Ministry of Health, and an opportunistic national newborn screening program is currently being launched on the back of an existing screening program for mother-to-child transmission of HIV ^16^. Nevertheless, most clinics within Uganda still offer little but folic acid and infection prophylaxis when available. They also do not have the capacity to monitor hydroxyurea with frequent clinical follow-up and laboratory tests, as is routinely undertaken in high-income settings and in current clinical trials evaluating the safety of hydroxyurea in the African region ^17, 18^.

Few studies have been published on the epidemiology of childhood SCD in sub-Saharan Africa. In recent years, we have shown that mortality is extremely high in undiagnosed and untreated children (50–90% by 5 years) ^12^, but is considerably lower in children who attend clinics and receive even the simplest treatments. For example, in 122 children with SCD recruited aged 3–12 months in Kilifi, Kenya, none of whom received hydroxyurea treatment, under-5 mortality was 29 (5.8 (95% CI 4.0–8.6) deaths/100 child-years (CY)) ^19^, yet it was significantly lower (2.9; 1.5–5.9/100CY) among those receiving a package of basic care, including education, antibacterial prophylaxis with penicillin V, and regular follow-up at a specialist clinic. Despite these reductions in overall mortality, the disease burden remained substantial, with an overall incidence of hospital admission rates for severe anemia, stroke, and invasive bacterial infections of 20.9 (17.3–25.2)/100CY, 4.8 (3.2–7.1), 0.7 (0.2–2.8), and 3.0 (2.0–4.7)/100CY respectively ^19^.

### Hydroxyurea

Hydroxyurea is orally administered once daily and has a well-established safety and efficacy profile when used to treat SCD in high-income countries with resources for regular monitoring. It is the only disease-modifying treatment for SCD treatment worldwide that is currently licensed by both the FDA and the EMA ^8^, and is also included for SCD treatment on the WHO list of essential medicines for children ^20^. Hydroxyurea increases levels of both fetal (HbF) and total hemoglobin, and reduces both inflammation and hemolysys ^8^. These therapeutic effects lead to wide-ranging benefits that include better growth, lower levels of end-organ damage, fewer strokes and painful crises and reduced mortality ^8^.

Generic tablets and capsule formulations of hydroxyurea have been manufactured and distributed in Africa by several companies. While a growing number of patients within the continent are now beginning to use hydroxyurea, several factors, including a higher prevalence of nutritional deficiencies and infectious comorbidities, make it unclear whether hydroxyurea will be as safe and effective in Africa as in high-income settings ^21^. Of note, in high-income countries, the standard approach to hydroxyurea therapy involves individually escalating the dose while closely monitoring hemoglobin parameters to a point approaching toxicity - the maximum tolerated dose (MTD) - in the belief that this will bring the greatest benefits ^8^. The need for frequent visits to clinics that are staffed by clinicians with expertise in the use of hydroxyurea and who have access to expensive laboratory monitoring using full blood and reticulocyte counts and HbF measurements, neither of which are routinely available in most hospitals within the public sector across Africa, together with the need for a range of different strength formulations to achieve an accurate MTD, mean that this individualised, dose-escalation approach, is unreachable for the majority of children with SCD who live in low-income countries in sub-Saharan Africa. Furthermore, until very recently, the only formulations of hydroxyurea that have been widely available in Uganda have been capsules of 500 or 200 mg strength, which do not easily provide the flexibility to provide sufficiently accurate dosing to achieve the desired MTD dosing range.

For these reasons, further trials exploring more pragmatic public health approaches to dosing, which can be used safely without the need for extensive routine laboratory monitoring, have increasingly been identified as major research priorities ^21– 24^. Current recommendations for hydroxyurea treatment under the Uganda Clinical Guidelines - frequent crises (>5/year), abnormal transcranial Doppler ultrasound velocities (which are not routinely measured within the country), stroke, and acute chest syndrome ^25^–are highly conservative, meaning that many of the potential benefits of early and universal implementation are likely to be missed.

### Relevant clinical trials

A recent Cochrane Systematic review of use of hydroxyurea in SCD treatment concluded that “there is still insufficient evidence on the long-term benefits of hydroxyurea, particularly in preventing chronic complications of SCD, recommending a standard dose or dose escalation to maximum tolerated dose” and that “future studies should be designed to address such uncertainties” ^26^. That review, however, included no data from Africa, where most children with undiagnosed and untreated SCD live.

Two small trials conducted in India (n=60 randomized to hydroxyurea versus placebo ^27^ and n=144 single group receiving hydroxyurea ^28^), found that low-dose hydroxyurea (10 mg/kg/day) was both safe and effective in terms of HbF responses. However, baseline HbF levels are higher in Indian patients than in African patients ^26^, indicating that these results are not necessarily generalizable to Africa. To date, only one placebo-controlled trial (n=208 (NO-HARM ^17^)), one single-arm, open-label trial (n=635 (REACH ^18^)), and one low-versus high-dose trial (n=220 (SPRING ^29^)) of hydroxyurea have been published in Africa, while a further single-arm, open-label trial (n=29 ^30, 31^) is currently in progress. None has been powered for mortality, and all have included levels of follow-up and routine laboratory monitoring that would be unrealistic for wider implementation in low-income settings. Several of the investigators involved in the current trial are involved in the REACH trial, through which we have already shown that hydroxyurea escalated to the MTD is both safe and effective against a broad range of clinical outcomes, including malaria episodes and death ^18^. However, the individualized dose escalation of hydroxyurea to the MTD in REACH requires intense levels of laboratory monitoring to titrate dosages, approaches that are not feasible broadly, and owing to the high costs of the laboratory tests, are currently not available for widespread rollout in low-income settings. In addition, four different formulation strengths were used to achieve precise MTD dosing, which was a key feature of the trial. As a result, it cannot be assumed that hydroxyurea would be equally safe or effective if introduced widely and at lower-level health centers, using standardized, and inevitably on average lower doses without similar intensive routine laboratory monitoring.

### Real world implementation

Where hydroxyurea is available in Africa, it is most often used informally and with little of the laboratory monitoring that is standard in treatment recommendations globally. Moreover, it is generally reserved for children with multiple SCD complications and is not widely recommended as a standard of care for all patients. While the REACH ^18^, NOHARM ^32^ and SPRING ^29^ trials have suggested that escalating hydroxyurea to the MTD is both safe and effective in Africa when used in the general population of children with SCD, rather than those with multiple complications, all three trials involved levels of clinical and laboratory monitoring that were similar to those used in high-income settings, leaving major questions about what might happen if hydroxyurea therapy was to be implemented under real-world conditions. Therefore, some experts have suggested that hydroxyurea should be prescribed at moderate doses (10–20 mg/kg/day) that can be safely used with minimal monitoring ^27, 28, 33, 34^. Conversely, others have proposed that higher doses should be used to maximize benefit ^8^, although it cannot be assumed that this will not be associated with an increased risk of harm from toxicity if implemented under real-world conditions. Therefore, a major aim of the current trial is to address this question through an open-label randomized comparison of high-dose (25 mg/kg/day, range 15–30 mg/kg/day) versus low-dose (10 mg/kg, range 6–13 mg/kg/day) hydroxyurea delivered pragmatically through weight-band-based dosing.

### Prophylaxis against other infections

Malaria and bacterial infections are widely considered to be the most common causes of early mortality among children with SCD in sub-Saharan Africa ^35^. Previous work from the trial investigators suggested that although the absolute risk of malaria may not be increased, it more often results in catastrophic anemia and subsequent death ^36, 37^. Moreover, we have recently estimated that the risk of bacteremia is exceptionally high, at approximately 5% per year ^38, 39^. Although *Streptococcus pneumoniae* was the most important organism in these previous studies, almost half of all infections were caused by gram-negative bacteria, including non-typhoidal salmonellae (NTS) (18%), *H. influenzae type b* (12%), *E. coli* (6%), and Acinetobacter sp. (6%) ^38, 39^.

Several drugs are commonly used for malaria prophylaxis in different parts of Africa, including chloroquine, proguanil, and sulfadoxine-pyrimethamine (SP), which has recently been recommended as the standard of care (SOC) in Uganda ^40^. However, given the current rates of resistance, all these drugs may now be considered sub-optimal ^37^. Similarly, although twice-daily penicillin V prophylaxis and the introduction of newer vaccines will almost certainly reduce the rates of infections caused by both *S. pneumoniae* and *H. influenzae* type b, it is possible that greater benefits would accrue with protection against a broader range of infections, the most important of which are outlined above. A recent major systematic review of antibiotics and antimalarials ^41^ in SCD identified a few studies, most of which were too small to make definitive conclusions.

Multi-drug resistant *P. falciparum* malaria is now common throughout much of sub-Saharan Africa and is a particular concern in Uganda ^42^, where the efficacy of the standard preventive regimen for children with SCD (monthly SP ^40^) is now <10% ^42^. We hypothesised that an alternative regimen, weekly dihydroartemisinin-piperaquine (DHA-PQP), may be more effective. Intermittent preventive treatment (IPT) with DHA-PQP is both highly effective and very well tolerated in infants, children, adults, and pregnant women ^43, 44^. Although DHA-PQP has been associated with QT prolongation, in a recent major review of over 200,000 individuals, the WHO found no evidence for a raised incidence of sudden cardiac death ^45^. While various dosing strategies have been used, a weekly approach has predicted benefits over other regimens, in particular higher efficacy and reduced concerns about compliance and safety, because of lower peak levels from a lower dose taken every week compared with a higher dose taken once a month ^46^. Nevertheless, recognizing that DHA-PQP might behave differently in children with SCD or in those on hydroxyurea, as well as factorially randomizing children between weekly DHA-PQP versus monthly SP, we will also conduct a sub-study to monitor both cardiotoxicity and pharmacokinetic (PK) and pharmacodynamics (PD) in a subset of trial participants. We will also monitor the possibility of emerging malaria resistance by using molecular methods.

In terms of antimicrobial prophylaxis, a range of potential agents have activity against a broader range of Gram-positive and negative organisms than penicillin V, including azithromycin, amoxicillin, co-amoxiclav and the cephalosporins. However, all pose risks in terms of side effects and the development of wider antimicrobial resistance in the commensal microflora. On balance, cotrimoxazole (CTX) strikes a good balance between risk, including the development of antimicrobial resistance, and potential benefits. Moreover, unlike many other options, CTX is not normally used for the treatment of serious and life-threatening infections. CTX is cheap, readily available, and widely used as prophylaxis in both HIV-exposed uninfected and infected children, where it has been found to be both extremely safe and strongly protective against all-cause mortality despite high background levels of microbial drug-resistance ^47– 49^. Finally, its once-daily dosing (as opposed to twice daily for penicillin) is attractive for compliance. Together, these characteristics indicate that the use of CTX rather than penicillin V could be easily implemented both quickly and at scale. Furthermore, the sustained increase in the risk of infections beyond the age of 5 years ^50– 52^ means that use throughout the whole of childhood could be a valuable alternative strategy for bacterial prophylaxis, compared with restricting antibiotic prophylaxis to those under 5 years as recommended in current guidelines ^25^. The main agent targeted by penicillin V is *S. pneumoniae*; however, widespread pneumococcal vaccination (in particular in all children enrolled in this trial) now makes this a less important pathogen to target with antimicrobial prophylaxis. We will, therefore, factorially randomize children to receive daily CTX (at all ages) versus twice daily penicillin V (to those under 5 years only). As both CTX and SP are sulphonamide-based and likely have similar impacts on both malaria and bacterial infections, children factorially randomized to the daily CTX and monthly SP arms of the trial will receive CTX only, and will not receive additional monthly SP, but will be analyzed as in the monthly SP group.

## Protocol

### Trial registration

International Standard Randomised Controlled Trial Number: ISRCTN15724013 (registered on 25/02/2020).

Pan African Clinical Trials protocol number: PACTR 202401802272880 (registered on 09/01/2024).

Protocol Version 5.0 Date 18th December 2024.

This protocol follows the SPIRIT guidelines ^53^.

### Justification for the study

SCD is a major cause of mortality and morbidity during childhood in many sub-Saharan African countries. The main triggers are related to the pathophysiological consequences of the disease itself and bacterial and malarial infections. The overarching question that we wish to address is whether long-term survival and quality of life among children living with SCD in sub-Saharan Africa could be improved through the pragmatic delivery of simple and affordable interventions.

A large pragmatic mortality-endpoint trial of SCD is needed for several reasons. Previous trials investigating hydroxyurea have not been designed and powered to investigate the survival of children with SCD living in malaria-endemic regions of sub-Saharan Africa ^17, 18^. Moreover, strategies for delivery based on uniform weight-band-based dosing were not investigated in the two most definitive trials conducted in Africa to date ^17, 18^ which, further involved hydroxyurea escalated to the MTD and did not involve clinically driven, pragmatic, laboratory monitoring approaches that could be generalizable across sub-Saharan Africa where, in the main, there is a lack of specialist laboratory services. Existing trials, therefore, have not provided evidence that is needed to influence policies regarding the safety and efficacy of wider drug uptake in regions where quality-controlled routine and frequent laboratory monitoring are not available. However, failing to tackle SCD survival because of infrastructure constraints could mean that many countries within the region will miss their Sustainable Development Goals (SDGs) both in terms of deaths averted and their promise that “no one must be left behind” ^54^. Second, with demographic transition ^55^ an increasing number of children with SCD will inevitably survive to require medical attention, and to become chronically unwell, develop long-term complications including strokes, and place a disproportionate burden on medical and blood transfusion services ^7^. Finally, SCD is recognized as a key health challenge by international agencies ^56, 57^ and by African governments, including Uganda ^16^. This is manifested by the recent introduction of selective early life screening in Uganda during recent years ^16^ and plans for universal screening in the near future. Without trials such as Hydroxyurea - Pragmatic Reduction In Mortality and Economic burden (H-PRIME), it would be impossible to develop regionally appropriate policies that could genuinely impact morbidity and mortality. Specifically, while hydroxyurea is now increasingly being used, it is not known whether it will be either well tolerated or effective in an informal way in which it is most commonly used.

The most important outstanding questions are therefore:

1) Would hydroxyurea therapy commenced early in life for all children diagnosed with SCD be well tolerated and effective in reducing mortality and morbidity in sub-Saharan Africa if used at fixed weight-band-based doses with minimal clinically driven (rather than regular and pre-planned routine) laboratory monitoring?

2) Can further reductions in mortality and/or morbidity be achieved through better approaches to preventing malaria and bacterial infections?

Combining investigation of the different interventions into one trial not only allows us to evaluate the role of hydroxyurea against different backgrounds of interventions that may be used in the next decade, but also allows us to answer the most relevant management questions efficiently in a single trial.

### Study hypotheses

There are three hypotheses being tested:

- [Randomization 1, R1] Oral hydroxyurea at a fixed weight-band-based high dose administered daily with clinically driven (rather than routine scheduled) laboratory monitoring, without titrating doses to the MTD, will reduce all-cause mortality compared with fixed weight-band-based low-dose hydroxyurea administered three times a week with clinically driven (rather than routine scheduled) laboratory monitoring.
- [R2] Enhanced antimalarial prophylaxis reduces malaria-associated hospitalization versus standard of care (SOC) (open-label).
- [R3] Enhanced antimicrobial prophylaxis reduces all-cause hospitalization versus SOC (open label).

## Objectives

### General objective

The primary objective of this trial will be to identify pragmatic, effective, safe, and acceptable interventions to reduce short- and long-term mortality and morbidity in children with SCD in sub-Saharan Africa.

### Specific objectives

1. To determine the efficacy of the strategies listed above on mortality, other measures of morbidity will be used (see below for specific primary endpoints for each randomization).
2. To determine the safety and tolerability of the strategies listed above.
3. To identify the most cost-effective interventions to reduce mortality and morbidity, and assess their budget impact.
4. To investigate the cardiac safety of DHA-PQP in children with SCD.
5. To investigate the resistance patterns of malaria parasites acquired by children to different forms of malaria prophylaxis.

## Methods

### Study sites

The trial is being conducted at four health facilities in Eastern Uganda: Mbale Regional Referral Hospital, Mbale (lead site providing laboratory support for patient monitoring and sample storage and local data and trial management), Soroti Regional Referral Hospital, Soroti, Atutur District Hospital, and Ngora Health Centre IV.

These centers all run large SCD clinics that manage a heavy patient load. Nevertheless, they have relatively limited resources in terms of staff and laboratory facilities, reflecting the clinical situation throughout most of sub-Saharan Africa.

### Study design

H-PRIME is a phase III multicentre 2 × 2 × 2 factorial randomized controlled trial designed to simultaneously evaluate three approaches to reduce the morbidity and mortality associated with SCD.

### Study population

A total of 1800 HbSS children aged 1 to 10 years inclusive.

### Inclusion criteria

1. Aged 1–10 years inclusive.
2. SCD diagnosed by rapid testing and confirmed by either high-performance liquid chromatography (HPLC) or isoelectric focusing (IEF) at a qualified laboratory.
3. Have received conjugate pneumococcal vaccination against Hib and *S. pneumoniae* (the study team will facilitate the vaccination of otherwise eligible but unvaccinated children who may then be recruited).
4. Carers willing and able to provide consent and to bring the child for follow-up visits, as demonstrated by either regular attendance at SCD clinics to date or attending two visits (one of which may be the screening visit) before randomization.

This age range is based on the mortality risks of SCD, practicality of weight-band-based dosing, ability of children to swallow divided tablets, and low probability of pregnancy among female participants, which could complicate the conduct of the trial.

### Exclusion criteria

1. Weighing <8 kg – exclusion pending gain in weight to 8 kg or more (the lower limit at which accurate dosing can be achieved with trial preparation of HDU).
2. A child who already meet the criteria for starting hydroxyurea in national guidelines (frequent crises (>5/year), known abnormal transcranial Doppler ultrasound velocities, stroke, or acute chest syndrome).
3. Hydroxyurea was already administered, defined as having been prescribed hydroxyurea at any time during the preceding 6 months, or having received hydroxyurea for more than 3 months.
4. Concomitant medications will be contraindicated with any of the trial medications (hydroxyurea, SP, DHA-PQP, penicillin V, and cotrimoxazole) (including, but not limited to, nefazodone, verapamil, rifampicin, isoniazid, and ethambutol).
5. A positive pregnancy test at screening or enrolment visits.
6. Known allergy to any of the randomized drugs used in the trial, that is, hydroxyurea, SP, DHA-PQP, penicillin V, or cotrimoxazole.
7. Known cancer.
8. Clinical history of previous or existing liver or renal diseases unrelated to SCD.
9. Known cardiac ventricular dysfunction or failure or a previous history of cardiac arrhythmias.
10. Known HIV (these children should receive cotrimoxazole prophylaxis and many will be receiving antiretrovirals that are contraindicated with one or more trial medications (zidovudine, amprenavir, atazanavir, indinavir, nelfinavir, ritonavir)).
11. Current participation in any other clinical trial of an investigational medicinal product.
12. The receipt of a blood transfusion within the preceding 3-month period (this will render the diagnostic confirmation of SCD unreliable; children may be rescreened after 3 months if they do not meet other exclusion criteria).
13. Presence of acute infection on the day of screening (e.g., symptomatic *P. falciparum* malaria, pneumonia, septicemia, meningitis, newly identified tuberculosis), such children may be enrolled after recovery from an acute infection if they do not meet other exclusion criteria.

## Sampling

### Sample size

The primary outcome for high-versus low-dose hydroxyurea randomization is all-cause mortality. Data from a range of recent relevant trials ^18, 29^ suggest that a range of mortality rates is plausible among children treated with low-dose hydroxyurea in Africa. Table 1 summarizes these rates and the power to show a range of plausible mortality reductions with either 80% or 90% power within a trial recruiting 900 children to each group, assuming a 5% loss to follow-up. This shows that a trial involving a total of 1800 children is well powered to show an approximate halving of mortality between the randomized hydroxyurea groups and to show smaller differences at the upper end of plausible rates within the low-dose group. While the detectable relative reduction will be slightly larger at lower event rates in the low-dose group, the detectable absolute change in risk remained above 1 per 100 child-years, that is, we should still be able to detect any benefits associated with high-dose hydroxyurea given with pragmatic clinical and laboratory monitoring that still saved at least one extra child’s life for every 100 children treated for one year.

**Table 1.** Power calculation.

Mortality rate (deaths / 100 person years) in the low dose group	Relative reduction detectable for the high dose group with 90% power	Absolute risk reduction	Relative reduction detectable with 80% power	Absolute risk reduction
2.5	51%	-1.3	45%	-1.1
3.0	48%	-1.4	42%	-1.3
3.5	45%	-1.6	39%	-1.4
4.0	42%	-1.7	37%	-1.5

The malaria / antimicrobial comparisons are not for mortality. 1800 children randomized to DHA-PQP versus SP (or CTX in those randomized to CTX with standard antimalarials) will provide 86% power to detect a 36% relative reduction from 4.7 to 3.0 malaria-associated hospitalisations/100CY respectively, assuming a 5% loss to follow-up. Similarly, 1800 children randomized to CTX versus penicillin V will give 87% power to detect a 20% relative reduction from 20 to 16 all-cause hospitalisations/100CY respectively, assuming a 5% loss to follow-up. These calculations are based on a reasonable assumption that CTX provides equivalent antimalarial cover to SP, based on concerns about SP resistance and CTX effectiveness ^58^. These calculations assume that hydroxyurea does not directly affect either of these endpoints (i.e., does not include an inflation factor). Allowing even as much as a 50% reduction in malarial and all-cause hospitalizations associated with hydroxyurea, our power to detect these same relative reductions associated with antimalarial and antibiotic randomization drops from >85% to 76% and 79%, respectively, but we retain >85% power to detect relative reductions of 40% and 22%, respectively, which are still clinically meaningful.

Interactions will be explored in the subgroup analyses. If R2 and R3 are treated as a 4-arm comparison, assuming two-sided alpha=0.017 to allow for three pairwise comparisons with SOC for antimalarial and antibiotic combined, 1800 children provide 80% power to detect a 51% relative reduction from 4.7 to 2.3 malaria-associated hospitalisations/100CY and a 29% relative reduction from 20 to 14.2 all-cause hospitalisations/100CY.

### Sampling procedures and methods

Eligible children are screened for potential recruitment into the trial from several sources.

1. Those currently under active follow-up for SCD at hospitals where the trial centers are located (>3000 eligible children were already registered when recruitment started).
2. Children admitted to the pediatric wards of the same hospitals with SCD that was diagnosed as part of targeted screening.
3. Children identified through the national SCD early life screening programme.

Children who are not already registered at one of the SCD clinics at the participating centers undergo a minimum 3-month pre-treatment screening and observation period to allow diagnostic confirmation of SCD status and to ensure that the families are able and willing to return to the clinic for regular follow-up. Following informed consent for screening, 4 mls of blood is being collected, 2 mls into EDTA for confirmatory SCD testing, full blood count and plasma storage, and 2 mls into a serum separator tube for renal and liver function tests. Testing for SCD uses a rapid diagnostic test (either the Gazelle ^TM^ mini-electrophoresis or another validated method) followed by confirmatory testing by either HPLC at the Mbale Clinical Research Institute (MCRI) or IEF at the Uganda Central Public Health Laboratory. Screening consent is being obtained explicitly for this sample, which will determine whether children are eligible for the trial. The schedule for research blood testing and case record form (CRF) completion at recruitment and follow-up are summarized as extended data under the DOI https://doi.org/10.7910/DVN/QUGG6P ^59^. Children screening positive for HbSS will be established on SOC following the Uganda Clinical Guidelines 2023 ^25^. This includes folic acid (5 mg daily), malaria (SP), and antimicrobial prophylaxis (penicillin V if aged <5 years) as described below. Care is further standardized by ensuring that all children have received all vaccines within the WHO Expanded Programme on Immunization schedule, receive anti-helminthic drugs where appropriate (every 3 months in children <5 years), and have been issued an insecticide-impregnated bed-net to help prevent malaria. Before randomization, children will have to attend at least two clinic visits, either following screening if they are newly attending the clinic or through previous follow-up at the local clinic.

All children formally screened for recruitment had a screening CRF completed and registered in the trial register. Consent is also being taken for the storage of screening blood samples and for diagnostic testing by molecular methods.

### Consent process

The child should be physically present together with a trial clinician in either the site clinic or the hospital ward at the time of randomization. Before randomization, the trial team should confirm the results of the confirmatory sickle test as showing HbSS as well as other inclusion/exclusion criteria. Carers and participants (where applicable) must confirm that they have read relevant patient information sheets. Written informed consent to enter into the trial and be randomized must then be obtained from parents/guardians of, or other persons with legal responsibility (including legal authorities) for the child, after explanation of the aims, methods, benefits, and potential hazards of the trial, and before any trial-specific procedures are performed or any blood is taken for the trial. Older children should assent trial participation. Signed trial consent/assent forms must be kept by the investigator and documented in the CRF, and a copy should be given to the participant or family.

It must be made completely and unambiguously clear that the participant (or parent or guardian of a child) is free to refuse to participate in any aspect of the trial at any time and for any reason, without incurring any penalty or affecting their treatment or that of their child.

For all children, the primary caregiver providing consent is asked to nominate one or two other carers (depending on local practice) who would be responsible for the child’s welfare in the event that they are unable to continue caring for them. While the carer who consented is alive, counselling of any other person who brings the child to the clinic is necessary to explain the trial, but re-consent is not needed. In cases where the carer who originally gave consent died, we are obtaining re-consent from an alternative nominated primary caregiver.

After consenting to trial participation (day 0), enrolment assessments are being performed. When possible, particularly if there is doubt about the place of residence of a child and his/her family, a map should be drawn indicating the place of residence. Where mobile phone numbers are provided for contact, they should be called from the clinic. Blood is being drawn for a full blood count, plasma storage, and DNA extraction [to test for other genetic modifiers, including known conditions such as α-thalassemia and G6PD-deficiency and as yet unknown modifiers using genome-wide and DNA sequence-based studies (2 ml EDTA tube)] and for renal and liver function tests (2 ml serum separator tube).

## Trial treatments

In total, 1800 children will be randomized 1:1 factorially to each of the following comparisons, as summarized in the Figure.

- [R1] Daily oral high-dose hydroxyurea versus three-weekly oral low-dose hydroxyurea (n=900 in each group).
- [R2] Weekly DHA-PQP versus monthly SP (SOC) (open label) (n=900 in each group).
- [R3] CTX, administered once daily throughout childhood, versus penicillin V administered twice daily until 5 years of age (SOC) (open label) (n=900 in each group).

**Figure.**
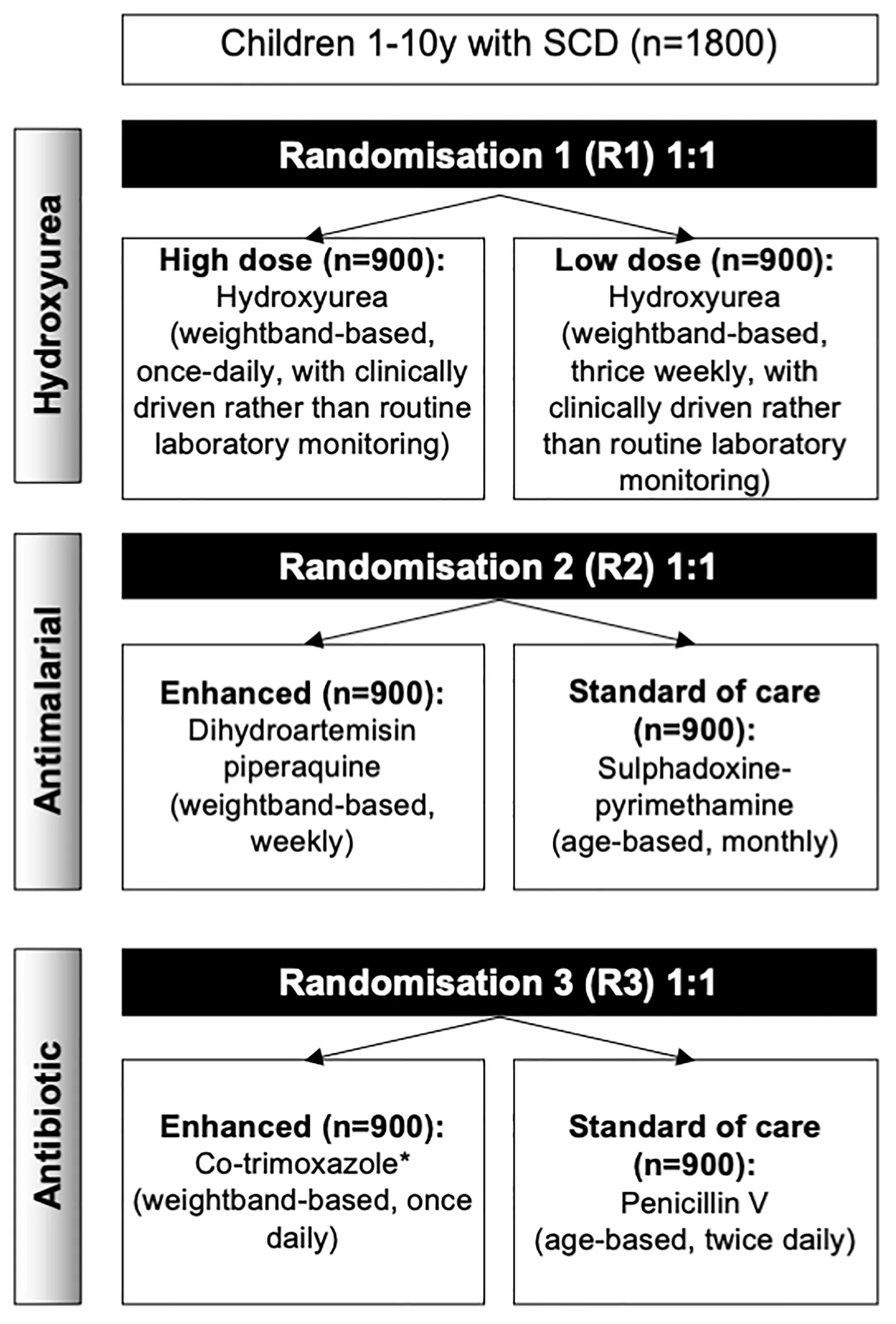
Trial schema. *Note: Because cotrimoxazole (CTX) and sulfadoxine-pyrimethamine (SP) should provide similar antimalarial cover and because both contain a sulfonamide component, which, with its long half-life, increases the risk of overdosing if both are co-administered, pragmatically, children randomized to both daily CTX prophylaxis and monthly SOC antimalarials (suphadoxine-pyrimethamine, SP) will receive daily CTX alone without additional monthly SP (with the further benefit of reducing pill count).

Both SOC groups are as defined in the Uganda Clinical Guidelines 2023 ^25^. Because CTX and SP should provide similar antimalarial cover and to avoid overdosing with sulfonamide compounds, pragmatically children randomized to daily CTX prophylaxis under antibiotic randomization, and to monthly SOC antimalarial drugs (SP) under the antimalarial randomization are receiving daily CTX alone without additional monthly SP (with the further benefit of reducing pill burden). Thus, R2 and R3 together compare four pragmatic strategies for improving antimalarial and antibacterial cover:

- Monthly SP (all ages) + twice daily penicillin V if <5 years (SOC).
- Weekly DHA-PQP (all ages) + twice-daily penicillin V for <5 years (improved antimalarial prophylaxis, equivalent antibiotic cover)
- Daily CTX (improved antibacterial prophylaxis while maintaining equivalent antimalarial cover)
- Weekly DHA-PQP + daily CTX (improved antimalarial and antibacterial prophylaxis)

All trial participants are being initiated on randomized open-label hydroxyurea at high (daily) or low (thrice weekly) doses and malarial and antimicrobial prophylaxis at standardized doses ( Table 2 and Table 3). The proposed interventions are all based on providing parts of tablets, reducing the complexity of procurement, and facilitating sustainability. Dosing is based on simple weight- or age-band-based algorithms, aligned with WHO thresholds and doses wherever possible. All children are also receiving 1 mg of folic acid daily. All children under five years of age are also receiving anti-helminthic drugs every three months. CRFs are being used to collect data on all the administered drugs.

**Table 2.** Weight-band based dosing of hydroxyurea, cotrimoxazole and DHA-PQP.

Weight band	Dose					
	High dose hydroxyurea		Cotrimoxazole	DHA-PQP		
	Tablets (1000mg)	Total mg	Total mg sulphamethoxazole /trimethoprim **	Tablets (20/160mg)	Tablets (80/640mg)	Total mg
3 - <6kg	*	-	100/20	1	-	20/160
6 - <8kg	*	-	200/40	1	-	20/160
8 - <10kg	¼	250	200/40	1½	-	30/240
10 - <11kg	¼	250	200/40	1½	-	30/240
11 - <14kg	¼	250	200/40	2 ***	½ ***	40/320
14 - <17kg	¼	250	400/80	2 ***	½ ***	40/320
17 - <20kg	½	500	400/80	3	-	60/480
20 - <25kg	½	500	400/80	3	-	60/480
25 - <33kg	¾	750	800/160	-	1	80/640
33 - <60 kg	1	1000	800/160	-	1½	120/960

Note: The low-dose hydroxurea randomization is to the same dose as the high-dose randomization but is given on only three days/week (Monday, Wednesday, and Friday) instead of every day of the week. * Children who drop below 8 kg temporarily discontinue hydroxyurea until they attain a weight of 8 kg or more. ** may be taken as pediatric tablets (400/80mg) or adult tablets (800/160mg) or parts of pediatric/adult tablets. *** given EITHER with one strength tablet or the other.

**Table 3.** Age based dosing of SP and penicillin V.

Age	Dose			
	SP		Penicillin V	
	Tablets (500/25 mg)	Total mg	Tablets (250 mg)	Total mg
1-<3y	½	250/12.5	½	125
2-<3y	½	250/12.5	½	125
3-<5y	½	250/12.5	1	250
5-<10y	1	500/25	-	-
10-<15y	2	1000/50	-	-

Body weight is being obtained by weighing the children on a standardized digital weighing scale at enrolment and every subsequent visit. The children are being weighed in light clothes without shoes. The body weight reported by the parents is not acceptable. This weight is being used to determine the correct weight band for the prescribed dose until the next visit. To reduce the risk of drug toxicity, doses issued until the next visit are always being based on the weight at the current visit, even though children may gain weight before their next visit. For example, a child weighing 9.9 kg at their current visit is prescribed the dose for the 6–<10 kg weight band, even though it is likely that they will increase their weight before their subsequent visit.

Patients and caregivers are being advised that all once-daily drugs should be taken together at a fixed time of day, either morning or evening, according to their preference, which can vary over time in the trial but should not vary day by day.

Randomization in each part of the factorial is being stratified by center, hydroxyurea initial dose (to ensure balance across the specific doses, Table 2), and the other randomizations in the factorial. Randomization is being performed by the Mbale Data Center (which has reliable power and internet connectivity) using an online randomization server. Other sites are telephoning the Mbale Data Center to perform randomization. Randomization lists were prepared by the Trial Statistician at the MRC CTU before the trial began, using random permuted blocks stratified by trial center and initial hydroxyurea weight band (or family, see below). These lists were then securely incorporated into the online randomization server to ensure allocation concealment. If there are connectivity issues, randomization is delayed because this is not an emergency situation.

We do not consider it ethical to restrict randomization to one child per family, so we are allowing multiple eligible individuals from the same family or household to enter the trial. However, where possible, we aim to randomize all children within the same family to receive the same allocation to all randomized interventions because the hydroxyurea randomization could prove confusing if different family members were assigned to different arms and received different dosing frequencies (some once-daily, some thrice-weekly). Also, with regard to the second and third randomizations, there is some risk that parents could confuse weekly DHA-PQP doses with monthly SP doses, or daily penicillin V or CTX allocations within families. While this would not be necessary if recruitment were restricted to one child per family, any such restriction runs the risk of drug pooling and dilution of any genuine treatment effects.

With these considerations in mind, we are catering to multiple children within the family. First, we are screening all children within families wherever any might be affected, such that randomization of all affected children within a family can occur simultaneously (i.e., per family randomization). We are including family randomization as an additional stratum in the weight-band-based dosing, since families will require more drug for multiple children (i.e., strata will be 3–<8 kg, 8–<17 kg, 17–<25 kg, 25–33 kg, 33 kg+, family). Finally, all participants are being counseled not to share their trial drugs with anyone who has not been recruited for H-PRIME.

### Trial medications and treatment schedule

***Hydroxyurea*.** Children are being randomized 1:1 to receive hydroxyurea at a fixed high dose versus hydroxyurea at a fixed low dose. Both are being provided as 1000 mg tablets (supplied by Novartis), which are scored three times into four 250 mg sections. This hydroxyurea formulation has been licensed recently by the National Drug Authority in Uganda.

We are delivering the drug at two different target doses: a high dose of 25 (15–30) mg/kg/day or a low dose of 10 (6–13) mg/kg/day using fixed weight-band-based doses, as shown in Table 2. The high-dose group are taking hydroxyurea every day while the low-dose group are taking hydroxyurea three times a week (suggesting Monday, Wednesday, Friday) to fit with the school/working week but this is flexible based on parent/carer preferences.

***Dose modifications and interruptions of hydroxyurea*.** H-PRIME will test the benefits and risks of pragmatically delivering hydroxyurea using weight band-based doses and administered only clinically driven hematological monitoring. Hydroxyurea dosing is being temporarily withheld for any participant whose weight drops below 8 kg during the course of the trial, until they re-attain a weight of 8 kg or more.

For research purposes, we are collecting standard data on hemtological efficacy and toxicity (full blood and reticulocyte counts, and HbF measurements) for 6 months; however, the results of these tests are not routinely being returned to clinicians in either randomized group and are not being used for clinical management. The only exception is if severely abnormal values are detected, as defined by grade 4 laboratory adverse events (AEs), as outlined in Table 4. Such results are being communicated to site clinicians as soon as they are identified. Under all other circumstances, decisions to interrupt the trial drug in both hydroxyurea randomized groups are being based on clinical grounds, supported where necessary by results of blood tests that would normally be available locally (i.e., hemoglobin only) and that are requested on clinical grounds only (based on signs/symptoms). Alternatively, if clinical complications coincide with phlebotomy visits, we are releasing the results of these scheduled blood counts based on specific clinical indications to limit the number of blood draws. However, reticulocyte counts and HbF tests that are not routinely available or affordable at the participating centers, or more widely in Uganda beyond a limited number of research centers, are never fed back to clinicians and should not be requested, as they are not used routinely for clinical management. We have previously used this type of approach successfully (and safely) in the large adult (DART ^60^) and pediatric (ARROW ^58^) trials of clinically driven monitoring for HIV treatment and informed management guidelines in Uganda.

**Table 4.** List of laboratory exceptions to the CTCAE version 5.0 grading.

Parameter	Grade 2	Grade 3	Grade 4
Hemoglobin (g/dL)	5.0 – 5.499	4.0 – 4.9	<4.0
Total white blood count (x 10 ^9^/L)	1.0 – 1.999	0.5 – 0.999	<0.5
Neutrophils (x 10 ^9^/L)	0.5 – 0.999	0.2 – 0.499	<0.2
Platelets (x 10 ^9^/L)	50 – 79	20 – 49	<20
Total Bilirubin ((mols/L)	85.5 – 171	172.7 – 342	>342
AST (IU/L)	150 – 300	301 – 1000	>1000
ALT (IU/L)	150 – 300	301 – 1000	>1000
Creatinine ((mol/L)	Doubling of baseline serum AND value ≥88.4μmol/L	141.4–176.8	>176.8
ARC (x 10 ^9^/L) (when Hb <7.0 g/dL)	50–80	10–49	<10

Clinicians are free to conduct hemoglobin tests for suspected severe anemia/crises or febrile illness, or send blood cultures for suspected bacterial sepsis, as locally available. If such a hemoglobin test, conducted on the basis of clinical suspicion, is <5 g/dl, in the first instance trial clinicians are advised to temporarily discontinue hydroxyurea treatment for a minimum of 1 week, and to carefully review such children clinically thereafter, with additional hemoglobin tests performed as indicated. In cases of persistent suspected toxicity to hydroxyurea and two or more temporary discontinuations of hydroxyurea, the primary course of action is either to restart hydroxyurea at half dose or discontinue the drug permanently. Additional management guidelines have been provided for the Manual of Operations (MOP). Toxicity is being managed in both randomized groups according to standard clinical practice.

Participants in H-PRIME are discontinuing low-dose hydroxyurea and being switched to daily dosing (equivalent to high-dose hydroxyurea) according to Table 2, if they develop any of the following criteria from the Uganda Clinical Guidelines 2023 ^25^ during the course of the trial:

1. >5 crises in one calendar year (i.e., six or more crises).
2. Known abnormal transcranial Doppler ultrasound velocities.
3. Stroke, as evidenced by the development of unilateral weakness or paralysis persisting for more than one week with features on neurological examination consistent with this diagnosis.
4. Acute chest syndrome. Given the difficulties in differentiating acute chest syndrome from pneumonia and the need for access to chest X-ray facilities, this is being operationalized by the occurrence of three or more admissions for pneumonia or lower respiratory tract infections within one calendar year.

***Expected toxicities of hydroxyurea*.** Hydroxyurea is expected to lower white blood cell, red blood cell, and platelet counts. This is common and, based on results from NOHARM ^17^ and REACH ^18^ (which together included more than 800 children treated for more than 3 years at MTD), is unlikely to reach dangerous levels that cause any problems. If a child presents with clinical symptoms suggestive of such toxicities, clinicians should request hemoglobin or other blood tests that are routinely available (see above). Other expected toxicities, according to the Summary of Product Characteristics (SPC), are summarized in Table 5. Many cases are mild and often resolve spontaneously.

**Table 5.** Expected adverse events on hydroxyurea from the SPC.

System	Frequency	Event
Blood and lymphatic disorders	Very common	Bone marrow depression, ^1^ including neutropenia, reticulocytopenia or macrocytosis ^2^
	Common	Thrombocytopenia, anemia ^3^
Nervous system disorders	Common	Headache
	Uncommon	Dizziness
Skin and subcutaneous tissue disorders	Common	Skin reactions (e.g. oral, ungula and cutaneous pigmentation) and oral mucositis
	Uncommon	Rash, melanonychia, alopecia
	Rare	Leg ulcers
	Very rare	Systemic and cutaneous lupus erythematous
	Not known	Cutaneous dryness
Reproductive system and breast disorders	Very common	Oligospermia, azoospermia ^4^
	Not known	Amenorrhea
Hepatobiliary disorders	Rare	Elevated liver enzymes
Gastrointestinal disorders	Uncommon	Nausea
	Not known	Gastrointestinal disturbances, vomiting, gastrointestinal ulcer, severe hypomagnesemia
Infections and infestations	Not known	Parvovirus B19 infection
Vascular disorders	Not known	Bleeding
Neoplasms	Not known	leukemia
General disorders	Not known	Fever
Other	Not known	Weight gain ^5^

^1^Hematological recovery usually occurs within two weeks of withdrawal of hydroxyurea. ^2^The macrocytosis caused by hydroxycarbamide is not vitamin B12 or folic acid dependent. ^3^Mainly due to an infection with Parvovirus or a splenic sequestration. ^4^Oligospermia and azoospermia are generally reversible but have to be taken into account when fatherhood is desired (see Section 5.3). These disorders are also associated with underlying diseases. ^5^Weight gain may be an effect of improved general conditions. Note: The frequency of complications, defined as very common (>1/10), common (>1/100 to <1/10), uncommon (>1/1,000 to <1/100), rare (>1/10,000 to <1/1,000), very rare (<1/10,000), and unknown (cannot be estimated from the available data), are based on the summary of product characteristics released by the European Medicine Agency ^61^.

## Antimalarial prophylaxis

### Products and treatment schedule

Children are being randomized 1:1 to enhance antimalarial prophylaxis with weekly DHA-PQP versus monthly SP (SOC, as defined in the Uganda Clinical Guidelines 2023 ^25^). Because CTX and SP should provide similar antimalarial cover, and to avoid overdosing with sulfonamide compounds, children randomized to CTX prophylaxis under antibiotic randomization and SOC antimalarials under antimalarial randomization are being prescribed CTX alone without additional SP (with the further advantage of reducing pill burden).

### Dihydroartemisinin-piperaquine (DHA-PQP)

DHA-PQP is being administered once weekly on a fixed day of the week, the day to be chosen by the primary caregiver (i.e., one-third of the monthly dose given once a week), following WHO-recommended doses (5–<8 kg 20/160 mg; 8–<11 kg 30/240 mg; 11–<17 kg 40/320 and ≥17 kg 60/480 ^43, 44, 46^) ( Table 2). This regime is simpler than the standard monthly DHA-PQP schedule, which involves dosing once a month on three consecutive days. Importantly, PK studies indicate that a once-weekly dosing regimen leads to lower peak levels ^46^ than a monthly dosing regimen. Although a recent meta-analysis of nearly 200,000 individuals ^62^ demonstrated no excess risk of sudden cardiac death with DHA-PQP, if there is any undetected excess risk, particularly in this SCD population, physiologically, it would be most likely related to maximum concentration. Hence, a weekly dosing regimen is highly likely to be safer from the point of view of potential cardiotoxicity. Furthermore, the weekly regime results in higher trough levels, which protect against the development of parasite resistance by reducing exposure to sub-therapeutic drug levels, and is also more tolerant to missing doses. We therefore consider that weekly dosing is highly likely to be both better tolerated and more effective than monthly dosing; this is supported by the Medicines for Malaria Ventures.

Following the dosing recommendations in the SPC, parents and participants are being instructed that the weekly dose of DHA-PQP should be taken on an empty stomach, with water but without food, for 3 h before and 3 h after a dose. For this reason, parents are advised to administer this weekly dose at a time that best fits their child’s routine. For example, it can be administered at least 3 hours after children have eaten breakfast, and children can then be limited to water thereafter until they receive their next meal at least 3 hours post-dose. Alternatively, it might be more convenient to administer the dose during the evening, at least 3 h after supper, and before children go to bed. Parents are being counselled regarding these two options and asked to follow the option they are most comfortable with for the duration of the study. As this is only a once-weekly activity, it is not anticipated that it will present an unduly onerous responsibility.

### Expected toxicities of DHA-PQP

The expected toxicities of DHA-PQP, along with their frequencies among children based on the SPC, are summarized in Table 6. Trial clinicians are being advised that the dose should not be reduced but that the drug can be discontinued if any of the general stopping rules described below in section “Protocol treatment discontinuation” apply.

**Table 6.** Expected adverse drug reactions that are associated with DHA-PQP in children.

System	Very common	Common	Uncommon
Infections and infestations	Influenza *P. falciparum* infection	Respiratory tract infection Ear infection	
Blood and lymphatic system disorders		Anemia Leukocytosis Leukopenia/neutropenia Thrombocytopenia	Hypochromia Lymphadenopathy Splenomegaly Thrombocytopenia
Metabolism and nutrition disorders		Anorexia	
Nervous system disorders			Convulsion Headache
Eye disorders		Conjunctivitis	
Cardiac disorders		Heart rate irregularities QT/QTc prolonged	Cardiac murmur Cardiac conduction disorders
Respiratory, thoracic, and mediastinal disorders	Cough		Epistaxis Rhinorrhea
Gastrointestinal disorders		Abdominal pain Vomiting Diarrhea	Nausea Stomatitis
Hepatobiliary disorders			Hepatitis Hepatomegaly Jaundice Abnormal liver function tests
Skin and subcutaneous tissue disorders		Dermatitis Rash	Pruritis Acanthosis
Musculoskeletal and connective tissue disorders			Arthralgia
General disorders and administration site conditions	Pyrexia	Asthenia	

Note: based on the SPC for Eurartesim ^63^. Events are listed under system organ class, and ranked by headings of frequency, the most frequent first, using the following convention: Very common (≥ 1/10), common (≥1/100 to < 1/10), uncommon (≥ 1/1,000 to < 1/100), rare (≥1/10,000 to <1/1,000), very rare (<1/10,000), not known (cannot be estimated from the available data).

### Sulphadoxine pyrimethamine (SP)

SP is given monthly following WHO-recommended doses (1–5 years ½ tablet (250/12.5 mg); 5–10 years 1 tablets (500/25 mg); 10–15 years 2 tablets (1000/50 mg) ^40^ ( Table 3).

### Expected toxicities of SP

At recommended doses, SP is generally extremely well tolerated; however, the following events rarely occur in individuals receiving SP:

- Skin reactions including rash, pruritis, photosensitivity and slight hair loss.
- Erythema multiforme Stevens Johnson syndrome and Lyell’s syndrome.
- Hepatic reactions including a transient rise in liver enzymes and hepatitis.
- Gastrointestinal reactions including nausea and vomiting.

Doses should not be reduced but the drug can be discontinued if any of the general stopping rules described below in section “Protocol treatment discontinuation” apply.

## Antimicrobial prophylaxis

### Products and treatment schedule

Children are being randomized 1:1 to enhance antibacterial prophylaxis with once daily CTX administered throughout childhood versus penicillin V administered twice daily until 5 years of age (SOC as defined in the Uganda Clinical Guidelines 2023 ^25^).

### Cotrimoxazole (CTX)

Cotrimoxazole dosing is following the WHO weight-band-based recommendations for prophylaxis in HIV-infected children: ^64^ 3–<6 kg 100/20 mg, 6–<14 kg 200/40 mg, 14–<25 kg 400/80 mg, and 25+ kg 800/160 mg ( Table 2). CTX may be administered as any formulation, including suspension 200/40 mg per 5 mls, 100/20 mg dispersible tablets, or 400/80 mg or 800/160 mg tablets or parts thereof. Dispersible tablets may be taken with water or mixed with feed. Children randomized to this arm of the trial will receive CTX throughout their trial participation.

### Expected toxicities of CTX

The expected toxicities associated with CTX are similar to those described for SP. Doses are not being reduced but the drug can be discontinued if any of the general stopping rules described below in section “Protocol treatment discontinuation” apply.

### Penicillin V

Penicillin dosing is being based on age: <3 years, 125 mg; 3-5 years 250 mg; both administered twice daily ( Table 3). Following the SOC in the Uganda Clinical Guidelines ^25^, children randomized to penicillin V will stop taking it at the age of five years.

### Expected toxicities of penicillin V

Penicillin V is usually well tolerated at these recommended doses. The most important events in children receiving penicillin V are as follows.

- Hypersensitivity including rash, pruritis, photosensitivity and in extreme rare cases anaphylactic reactions.
- Gastrointestinal reactions including nausea, vomiting, glossitis, and stomatitis.
- Blood dyscrasias including leucopoenia, thrombocytopenia and agranulocytosis.
- Overgrowth of normal commensals including candida.

Clinicians are advised not to reduce the dose but penicillin can be discontinued if any of the general stopping rules described below in section “Protocol treatment discontinuation” apply.

## Clinical assessment

All participants will be followed up for a minimum of 2 years and a maximum of 4 years. Trial follow-up will be completed 4 years after the first participant was randomized on 16 ^th^ January 16, 2024. All participants are being examined at the trial center clinics at screening, randomization, 1 and 3 months later, and then every 3 months thereafter. Visits include evaluations from both trial clinicians and nurses.

Trial visit schedules are being prepared for each participant at randomization, and participants are then being followed up using these schedules, even if their trial medication has been discontinued for any reason. The target dates for the trial visits are being determined by the date of randomization and are not affected by subsequent events. Clinics may choose to re-schedule visits to allow for public holidays or other unavoidable circumstances that affect the scheduled visit date, but the re-scheduled visit should ideally be no more than seven days (either before or after) from the originally scheduled visit date.

Participants are expected to attend the scheduled day unless an alternative date has been agreed in advance with the clinic. Participants are given a card with contact details for the trial research team so they can contact them if needed to rearrange a visit. If they are unable to attend the day, every effort should be made to complete the visit within seven days of the scheduled date. If a scheduled visit is missed without notice, then the clinic should endeavor to contact the participant by phone or home visit.

If a participant is more than seven days late for a scheduled trial visit, up to three attempts are made to contact and trace the child. An additional visit is performed as soon as possible, including the appropriate assessments specified in the trial schedule for the week of visit that was missed.

The schedules define visit dates (with windows) necessary for data collection, but the participant may be seen more frequently than needed at the discretion of the treating clinician, for example, if they develop side effects or other clinical events. At any such unscheduled visits, routine assessments for a standard clinician and nurse visit are performed (as for month 3). Other laboratory tests are also being performed as clinically indicated. In particular, participants in all groups may undergo all necessary diagnostic tests that are locally available for the clinical management of illness.

The participants’ caregivers are being encouraged to contact trial clinicians in the event of illness. In addition, hospital wards are being regularly reviewed by trial participants, and out-of-hospital mortality is being ascertained by telephone contact and community follow-up.

At each assessment (months 0, 1, 3, 6, 9, 12, 15, 18, 24, 27, 30, 33, 36, 39, 42, 45, 48), the following are being undertaken:

1. Administration of a symptom checklist by a nurse or other trial staff member to detect intercurrent illnesses or AEs related to medication. The severity and likely relationship of any AEs to medication are being documented by a physician/medical officer in the child’s notes and where applicable to the CRFs.
2. Medical history since the last visit included AEs (including specific questions about specific complications, including strokes and painful crises), signs and symptoms of SCD, and inpatient and outpatient visits, both with appropriate laboratory forms completed to document test results and blood transfusions.
3. Weight, height, mid upper arm circumference (MUAC)
4. Assessment of adherence to trial medication by pill counts and nurses administered a brief questionnaire (not Week 0).
5. EQ-5D-Youth assessment of daily living by a nurse or other trial staff member.
6. Changes in trial drugs and other concomitant medications and the use of clinically driven laboratory monitoring to determine any changes.
7. Malaria RDT, thick and thin films, and 2x filter paper whole blood samples for malaria resistance testing are being collected from children with documented fever (digital axillary temperature ≥ 37.5°C) and/or other symptoms suggestive of malaria along with a blood culture where possible. Children with signs warranting hospital admission are immediately referred. This is also being done at extra (non-scheduled) visits, where the child presents with fever.
8. 2x filter paper whole blood samples for pharmacokinetic analysis are being taken at any scheduled or unscheduled visit where any cardiac problems were reported (including suspected or proven cardiac arrhythmia). Children with signs warranting hospital admission are immediately being referred.
9. Money for transport is being given by the clinical coordinator or designee.
10. Urine pregnancy tests are carried out at every visit in any female who has reached menarche; any such female is counselled against becoming pregnant.

At specific visits, the following are undertaken:

1. At 0, 3, 6 and then 6-monthly, a full blood count is being performed together with HbF and reticulocyte counts. Other hematology (or laboratory) tests are also being performed if clinically indicated but are not required by the protocol. For example, HbF and reticulocyte counts may not be required for clinical management.
2. Annually, we are also administering the PedsQL measurement model for the Pediatric Quality of Life Inventory (self-reported for children >5 years and Parent Proxy Report for younger children), which has been widely used in HIV and other trials, along with the sickle modules of the same instrument ^65^
3. Annually, 4ml blood is being taken for plasma storage.

### Treatment data collection

Information about all medications received, including formulation, frequency, dose, and reasons for change, are being collected using the trial CRFs. All oral, intravenous, intramuscular, and topical treatments for any condition are being considered as concomitant medications, including blood transfusions.

### Non-trial treatment

All necessary concomitant medications are allowable in the trial. If a medication with a known drug interaction with one of the trial medications is essential for a participant's management, then if appropriate dose adjustment is not possible, the trial medication will be stopped and the concomitant medication used.

In a specific sub-study (see below), the following are also being undertaken:

1. Electrocardiography: enrolment (baseline), month 1 (pre-and 4 hours post-dose), month 6 (pre-and 4 hours post-dose), and month 12 (pre-and 4 hours post-dose), and then annually.
2. Pharmacokinetics: In the same children at the same time points, blood on filter paper are being collected for retrospective analysis of DHA-PQP and SP blood levels.

### Procedures for assessing efficacy

1. Clinical events (all participants).
2. Survival status is being recorded at each visit. Any participant who missed a scheduled clinic visit without withdrawing consent is being traced for their vital status.
3. Other serious adverse events (SAEs) are being reported as soon as the trial staff become aware of them (see below), including through ward rounds for admitted children. Given the catchment areas of hospitals, children are generally re-admitted to the same clinical site. The details reported include laboratory data and additional clinical narratives.
4. During all visits, trial personnel are soliciting for any history of intercurrent hospital admissions and/or blood transfusions.

### Hematological recovery (all participants)

Hemoglobin, HbF, and other blood count parameters are being measured every six months. The results of these blood tests are not being released to clinicians unless clinical complications coincide with phlebotomy visits and the clinician requests a hemoglobin test result for clinical reasons (based on signs/symptoms). The only exception is the detection of potentially dangerous abnormal values, as defined by grade 4 laboratory adverse events ( Table 4), which are automatically returned to clinicians.

### Procedures for assessing safety (all participants)

The symptom checklist used at each visit explicitly prompts for symptoms that are related to possible drug toxicities. Additional safety blood tests or investigations that are available locally may be performed to investigate symptoms or monitor emergent laboratory test abnormalities, as clinically indicated. Serious, solicited, and grade 3 or 4 adverse events are being reported using dedicated CRFs.

### Procedures for assessing adherence (all participants)

Adherence to all intervention drugs is being assessed in all participants at each visit by pill counts for tablets and a nurse-administered questionnaire to the child’s caregiver or, where appropriate, to the child (at the discretion of the nurse or doctor, depending on age).

### Pregnancy

Hydroxyurea is a Category D drug with a teratogenic potential. There are no adequate and well-controlled studies in pregnant women; therefore, precautions are being taken in the H-PRIME trial to recommend against pregnancy and to regularly monitor menstruating female participants for pregnancy. Only children under 11 years of age are being recruited; given the pubertal delay associated with SCD ^66^, there is a low likelihood that such girls will already have reached menarche. Post-randomization, whether a girl has reached menarche, is being ascertained at every visit. All girls with childbearing potential are counselled about the potential risks associated with pregnancy while taking hydroxyurea and the potential risks to the infant. Upon reaching menarche, girls are permitted to choose to discontinue hydroxyurea and continue to receive other trial medications and follow-up in the trial, if they wish. Girls who wishes to continue on hydroxyurea, are advised to use highly effective contraception if sexually active, and to report to the study staff as soon as possible if there is a possibility of pregnancy. Pregnancy testing is conducted at every visit for any girl who has reached menarche. Hydroxyurea will immediately be discontinued in any girl who is found to be pregnant. Any pregnant participants will receive counselling about their options and support to make decisions about continuing pregnancy. Pregnancies will be followed up to determine the outcomes and status of the mother and child. Pregnancy complications will be reported as an AE or SAE, as appropriate; pregnancy per se will not be reported as an AE or SAE. Any SAE occurring in association with a pregnancy brought to the investigator´s attention after the participant has completed the study and considered by the investigator as possibly related to any investigational medical product will also be reported as SAE.

Male participants will also be counseled about the need to avoid fathering a child while taking hydroxyurea.

### Trial withdrawal

In consenting to the trial, patients will have consented to trial treatment, data collection, and follow-up. If a carer wishes to withdraw their child from trial treatment, the investigator will explain the importance and benefits of follow-up and the value of allowing routine clinical data to be used for trial purposes. A parent or guardian who chooses to discontinue trial treatment for their child will be encouraged to follow other trial procedures and follow-up schedules. However, if they do not wish to remain on trial follow-up, their decision will be respected and the participant will be withdrawn from the trial completely. The MCRI will be informed of this in writing, using appropriate documentation. Prior to transferring out of the trial, the parent or guardian will be asked to perform assessments as appropriate for the final trial visit. They will be free to refuse any or all the individual components of the assessment.

As consent cannot be withdrawn for data already collected, if follow-up is stopped early, the medical data collected during their participation in the trial up to this point will be retained and used in the analysis. Similarly, samples obtained prior to this time will be processed according to the protocol unless the parent or guardian explicitly and unprompted requests otherwise. Consent for future use of stored samples can be refused when leaving the trial early (but this should follow a discussion). If consent for the future use of stored samples already collected is refused, then all such samples will be destroyed following the policies of the institution.

Any participants who do leave the trial will subsequently be allowed to reconsent if they change their minds about participation. If they do return, they will follow the same randomization as initially allocated. Any participants who stop trial follow-up early will not be replaced (the sample size calculation includes adjustment for loss to follow-up).

### Protocol treatment discontinuation

The following is a general list of reasons for discontinuation of the randomized treatment. In consenting to the trial, parents/guardians consented to trial treatment, trial follow-up, and data collection for their child. However, an individual participant may stop any one or all of their randomized trial treatments early, or it may be stopped early, either temporarily or permanently, for any of the following reasons:

1. Unacceptable toxicity or adverse event.
2. Intercurrent illness that prevents further treatment.
3. A fall in weight to less than 8kg.
4. Any change in the participant’s condition that justifies the discontinuation of the trial treatment, in the clinician’s opinion.
5. Inadequate compliance with the protocol treatment in the judgement of the treating physician.
6. Withdrawal of consent for treatment by the participant or their carer.
7. The onset of pregnancy in female participants.

As participation in the trial is entirely voluntary, participants are allowed to choose to discontinue the trial treatment at any time without penalty or loss of benefits, to which they are otherwise entitled. Although the participants will not be required to provide a reason for discontinuing their trial treatment, a reasonable effort should always be made to establish this reason while fully respecting participants’ rights.

### Trial products, storage, and accountability

For all trial drugs, the designated trial pharmacist confirmed the receipt of supplies before the trial started. Inventories are conducted undertaken regularly and logs are being returned to the MCRI. All drugs dispensed to the participants are being recorded in a dispensing log. At each site, a trial pharmacist must maintain complete records of all the medications dispensed and returned. Carers are provided with a full supply of drugs at each visit, which is sufficient to last until the next scheduled clinic visit. All drugs are open-label and are, therefore, being prescribed in exact amounts until the next clinic visit. No additional pills of these open-label drugs are being dispensed to avoid caregivers returning to the clinic when these drugs are finished. Carers are being requested to return all empty bottles and any unused drugs to the follow-up clinic. No drug assigned to a participant should be used by anyone else. Unused trial drugs must be returned to the site if a participant withdraws from treatment.

### Accountability and unused drugs

The procedures for drug distribution, labelling, accountability, and destruction are detailed in the H-PRIME Pharmacy MOP. Drug accountability is regularly monitored and the remaining stocks are checked against the amounts dispensed. At the end of the trial, all remaining investigational drugs will be destroyed. The MCRI centrally monitors drug accountability and during site visits.

### Compliance and adherence

The 2023 Ugandan Clinical Guidelines for SCD recommend daily penicillin V prophylaxis (in those aged <5 years), folic acid (all ages), and monthly SP (all ages) in children with SCD. Therefore, we do not anticipate substantial compliance problems in this group with motivated caregivers, given their child’s chronic condition. Trial drugs are being used relatively widely as prophylaxis in children, with modest rates of toxicity and high acceptability. As the interventions are being started immediately following randomization, suitable patient information and fully informed consent procedures ensure that the participants understand the trial requirements. During clinic visits, all caregivers are being asked questions about adherence using a standard questionnaire. Any non-compliance due to lack of acceptability or side effects would also likely occur if the interventions were incorporated into clinical practice and are part of a pragmatic strategy being evaluated. The intention-to-treat comparison will therefore incorporate such “strategy noncompliance, which is anticipated in general clinical practice. Finally, in our current and recent REACH ^31^ and NOHARM ^17^ trials, compliance with daily hydroxyurea therapy escalated to MTD was universally high, reaching >95% across both trials and all trial sites (Russell Ware, personal communication).

### Trial medication overdose

Parents or guardians of the children participating in the trial are being counselled about the importance of taking oral medications exactly as prescribed. They are being asked to contact the H-PRIME clinic immediately if their child is overdosed to receive appropriate advice. Participants will then be managed on a case-by-case basis, and toxicity will be managed in all randomized groups according to standard clinical practice.

## Sub-studies

### Health economics

Little is known about the economic burden faced by families with children affected by SCD, particularly in sub-Saharan Africa. Policymakers require information on the costs and health effects of alternative interventions when considering how to allocate limited resources to meet a population’s health needs. Most research on the economic aspects of SCD has focused on the medical costs borne by insurance or healthcare providers in the United States ^67^, although three recent studies did originate from Africa ^68– 70^. As part of this trial, on conclusion of the trial we will therefore include an analysis of the cost-effectiveness of the three interventions.

To reduce the burden on families, we will calculate the cost-effectiveness of the intervention primarily using a healthcare provider perspective, but also considering the time children spent off school. Unit cost data will be obtained from basic costings, costs available in the literature, and data collected in other economic analyses of management strategies for children in Uganda (e.g. FEAST ^71^, TRACT ^72^ and COAST ^73^). The association between costs, benefits, and baseline characteristics will be estimated, and cost-effectiveness is estimated to be subject to uncertainty over the model parameters. Long-term extrapolation outside the trial is based on stochastic compartmental models. As mortality is the primary outcome, cost-effectiveness will also be estimated based on the number of lives saved. Finally, we will also study affordability by relating the distribution of cost per child to that of family income and healthcare expenditure per child in the area.

### Cardio-safety and pharmacokinetics

Although DHA-PQP is associated with QT prolongation, a major review of over 200,000 individuals from the WHO found no evidence for a raised incidence of sudden cardiac death ^45^. As above, a weekly DHA-PQP dosing approach has predicted benefits over other regimens, including higher efficacy and reduced concerns about compliance and safety, because of lower peak levels from a lower dose taken every week compared with a higher dose taken once a month ^46, 74^.

However, to assess cardiotoxicity in children with SCD, 200 children enrolled in H-PRIME at the Mbale site will provide optional consent to enter a cardiac and pharmacokinetic substudy. At enrolment (before their first dose), all children will undergo a fully automated 12-lead ECG reading. Any abnormal results will be confirmed through manual review of these ECGs, with a particular focus on the QT intervals and QTcF (corrected QT intervals, using Fridericia’s correction) ^75^. This will be repeated at one month, 6, and 12 months, and annually thereafter. At post-randomization visits, children involved in this sub-study are being asked to visit the clinic on the weekday that they would usually take their DHA-PQP dose if randomized to DHA-PQP, or on the day they would take their monthly SP if randomized to the antimalarial control (or scheduled day if randomized to CTX). 12-lead ECGs are being recorded both pre-dose and 4h post-dose. Those in the DHA-PQP group take their drugs on an empty stomach with water only because drug absorption is less predictable if taken with fatty foods. The primary endpoint of this sub-study will be the change in QTcF from baseline to 4h post-dosing at 1-year post-randomisation. Secondary sub-study endpoints will include the QTcF change at other time points, the number with QTcF prolongation of >60 ms from baseline at each subsequent visit, and the percentage with QTcF intervals of >500 ms at any time point. Endpoints based on QTcB will also be secondary outcomes, as both correction factors introduce artifactual prolongation or shortening, particularly in children with tachycardic heart rates ^75^.

Including 200 children in the sub-study (expected to be equally distributed between DHA-PQP versus antimalarial control by design, and similar to all other randomizations), provides at least 90% power to detect differences of 10 ms between DHA-PQP and antimalarial, assuming an SD of 20 ms (slightly larger than in a previous study conducted in Papua New Guinea ^76^, given that children with SCD are younger) and 20% without valid readings at 1 year. Recruiting 100 children receiving DHA-PQP would provide a final 95% confidence interval around an observation of no QTcF intervals of >500 ms during the trial from 0–1.8%, it could reliably exclude this clinically relevant outcome in >2% of children. The independent Data Monitoring Committee (DMC) also monitors unblinded outcome data from this cardiac substudy in regular meetings.

In order to relate any changes in QTFc to drug levels, at the same post-randomization time points, we are taking whole blood filter papers from the sub-study children receiving DHA-PQP pre-dose (to reflect a trough level) and at 3-4 hours post-dose (to reflect a peak level). Piperaquine levels will then be determined retrospectively at the Mahidol Oxford Research Unit in Bangkok.

### Molecular diagnostics and malaria resistance

We are collecting dried blood spots (DBS) during febrile events to investigate bacteremia, malaria, and malaria resistance profiles using molecular methods. Results from this work will be fed back to the study investigators within six months of submission for genotyping, and the results of these studies will also be monitored by the DMC. Additionally, filter paper samples are being collected from children on DHA-PQP to test for DHA-PQP levels during fever episodes. This is in addition to the routine testing for malaria using RDTs and thick and thin blood films.

### Long-term storage

Study samples are currently being stored at the MCRI, and the majority of the assays will be conducted at laboratories in Mbale and Soroti. However, if necessary, study samples may be shipped to a laboratory outside Uganda for testing; participants are informed about this, and explicit consent for this is obtained. Only enough of the sample will be shipped to complete the required tests. Once these tests have been conducted and the specific study has ended, any samples left over in that laboratory will be destroyed or returned to Uganda for long-term storage. Any researcher who wants to use study samples for a new study not included in this protocol will need approval from the Mbale Regional Referral Hospital Research and Ethics Committee and Professor Olupot-Olupot; again, participants are informed about this and explicit consent for this obtained.

## Trial outcome measures

### Primary outcomes

- R1: Mortality (all-cause).
- R2: Malaria-associated hospitalization (diagnosed by rapid diagnostic test (RDT) OR microscopy OR PCR).
- R3: Hospitalisation for any reason.

All endpoints occurring from enrolment to the common trial follow-up end date 48 months after the first randomization will be included.

We selected all-cause mortality as the primary endpoint for the hydroxyurea comparison because of the challenges in ascertaining cause-specific mortality in the low-income settings where this trial is being conducted; it is arguably the most relevant endpoint both to patients and Ministries of Health in Africa from the perspective of resource allocation. However, the impact of improving antimalarial and antimicrobial prophylaxis is more likely on hospitalization than mortality, given the success of treatment, and this would still have a large impact on both families and the health system. Malaria-associated hospitalizations can be identified through various laboratory testing methods and hence are the primary endpoint for R2. However, blood cultures are extremely insensitive for identifying bacterial infections; hence, the primary endpoint for R3 will be all-cause hospitalization.

### Secondary outcomes

- Mortality (where mortality is not the primary outcome for that randomisation).
- Malaria-associated hospitalisations (where not the primary outcome).
- All-cause hospitalisations (where not the primary outcome).
- Any of the following SCD-specific complications requiring medical intervention (grade 2 or above): painful crisis, hand-foot syndrome, splenic sequestration, acute chest syndrome, or stroke
- Number of blood transfusions and volume of blood transfused.
- Hemoglobin (Hb) and fetal hemoglobin (HbF) concentrations.
- Grade 3 or 4 renal (creatinine) or liver function test (AST/ALT) results.
- Febrile events treated with intravenous antibiotics.
- Specific bacterial bloodstream infections confirmed by blood culture or molecular typing.
- Serious adverse events (SAEs).

### Other outcomes

- The total number of hospitalisations lasting 7 days or longer.
- The total duration of all hospitalisations.
- Febrile events reported at follow up visits or detected during hospitalisation.
- Grade 3 and 4 adverse events (AEs) further categorized according to whether they were potentially related to SCD ( Table 7).
- Grade 3 and 4 adverse events (AEs) considered possibly or definitely related to hydroxyurea or malaria/bacterial prophylaxis (for each specific randomization).
- Measures of nutritional status (including Z-scores for weight, height, and mid upper-arm circumference (MUAC)).
- Quality of life (QoL).
- Costs and cost-effectiveness.

**Table 7.** Recognised complications of SCD.

Acute chest syndrome	Empyema	Pain, long bone
Adeno-tonsillar disease	Hand-foot syndrome/dactylitis	Pain, severe abdominal
Albuminuria	Headache	Pain, sternal or rib
Amenorrhea	Hematuria	Priapism
Anemia (severe)	Hemiplegia	Proteinuria
Aplastic crisis	Hemolysis	Pneumonia
Arthralgia	Hepatic sequestration	Pulmonary embolism
Avascular necrosis of hip/shoulder	Hepatomegaly	Pulmonary hypertension
Bacteremia	Hospitalisation >24 hours	Pulmonary infiltrate on chest x-ray
Bone infarction	Hyperbilirubinemia	Pyelonephritis
Cardiac arrhythmia	Hypersplenism	Renal failure
Cardiomegaly	Hypertension	Renal insufficiency
Cerebrovascular accident	Hypocalcemia	Renal papillary necrosis
Cholecystitis	Hyposthenuria	Reticulocytopenia
Cholelithiasis	Hypotension	Reticulocytosis
Cognitive dysfunction	Hypoxemia (PO _2_ <65mm Hg)	Retinopathy
Constipation	Ileus	Retinal hemorrhage
Cranial nerve palsy	Infection, bacterial	Rhabdomyolysis
Death	Infection, pneumococcal	Seizure
Decreased renal function	Infection, line	Septicemia
Decreased lung function	Infection, viral	Silent organ infarction
Delayed growth/puberty	Jaundice	Skin ulcer
Depression	Leukocytosis	Splenic sequestration
Dizziness	Meningitis	Splenomegaly
Electrolyte imbalance	Nephropathy	Stroke
Elevated urinary urobilinogen	Osteomyelitis	Transient Ischemic Attack (TIA)
Elevated serum transaminases	Pain, back	Tonsillar enlargement or infection
Elevated TCD velocities	Pain, chest	Transfusion, unanticipated
Fever	Pain, joint	Vaso-occlusive pain

Note: List of expected and potentially serious adverse events associated with SCD. However, the table may not be inclusive. Investigators should use clinical judgment along with consensus knowledge regarding SCD to determine event expectedness.

## Interim reviews

During the trial, the DMC will meet at least annually to review unblinded data for all three randomized comparisons on enrolment, safety, adherence to randomized strategies, efficacy, and safety in strict confidence. The DMC met six months after the first recruitment and will subsequently determine the frequency of their meetings, which can be more frequent than annually, if necessary.

A decision to discontinue recruitment in all participants or in selected subgroups will be made only if the results are considered likely to convince the general clinical community and participants in the trial. The DMC will report to the Trial Steering Committee (TSC) if, in their view, the data provide proof beyond reasonable doubt that one of the allocated strategies is better than its comparator in terms of the primary outcome. The guiding statistical criterion for “proof beyond reasonable doubt” is the Haybittle-Peto criterion of a difference of at least three standard deviations in an interim analysis of a major endpoint. The TSC will then decide whether to amend (including the possibility of dropping one of the transfusion strategies) or stop the trial before the end of the planned follow-up. If a decision is made to continue, the DMC will advise on the frequency of future reviews of the data based on accrual and event rates.

## Trial monitoring

This trial is being monitored according to a Monitoring and Quality Management Plan, which sets out the frequency of visits, degree of source document verification against CRFs, and requirements for triggered on-site monitoring visits. This plan also details the procedures for reviewing and signing the monitoring reports. Monitoring is following the principles of ICH GCP and started with 100% source document verification, as in the previous TRACT trial. This will be reviewed for each site once a satisfactory and sustained quality assurance performance is established.

The trial-monitoring team consists of locally commissioned clinical trial monitors. The KWTRP CTF and the MRC CTU oversee the standards and quality of all trials that they conduct, and through their monitoring systems and SOPs are organized to ensure that all sites can be monitored with equal independence and rigor. All monitors are qualified and appropriately trained.

At each monitoring visit monitors:

- Verify completeness of the Trial Master File.
- Confirm adherence to protocol.
- Review eligibility verification and consent procedures.
- Look for missed clinical event reporting.
- Verify completeness, consistency and accuracy of data being entered on CRFs.
- Evaluate drug accountability.
- Provide additional training as needed.

The monitors require access to all patient medical records, including, but not limited to, laboratory test results and prescriptions. The investigator (or delegated deputy) works with the monitor to ensure that any problems detected are resolved.

Participating investigators agreed to allow trial-related monitoring, including audits, ethics committee review, and regulatory inspections, by providing direct access to source data and documents as required. Participants’ consent for this is also being obtained. Such information is treated as strictly confidential and will not be made publicly available.

The following data should all be verifiable from source documents:

- Signed consent forms.
- Dates of visits, including dates, and any trial specimens that have been obtained and processed in the laboratory.
- Eligibility and baseline values for all participants.
- Participant clinical and laboratory data.
- Clinical endpoints.
- Serious adverse events and severe (grade 3/4) adverse events (SAEs).
- Dates drug dispensed and (if necessary) drugs returned.
- Pharmacy/clinic drug logs.
- Concomitant medication dispensed.

Not all such information is being monitored; rather, the monitoring plan describes a risk-based approach to monitoring based on ongoing random samples of participant clinical and laboratory data, which may increase if issues are identified.

### Data management

All clinical and laboratory data are being recorded in the CRF and stored using a unique serial number identifier. The data are being entered into the REDCap database. All data are regularly backed up, and backup copies are stored both on and off. The paper records are being archived in locked cabinets that have limited access to authorized individuals. All data will be de-identified using a trial number prior to the presentation or publication of results. At the end of the trial, archived documents will be sent for long-term storage (10 years) at an appropriate facility according to national policies.

### Confidentiality

We are following the principles of the UK Data Protection Act 2018. In particular, the investigator must ensure that participants’ anonymity is maintained and that their identities are protected from unauthorized parties. Participants are assigned a trial identification number that is used on CRFs; participants will not be identified by their name. The investigator securely maintains a separate participant trial register linking these identification numbers to name and age. This unique trial number will identify all laboratory specimens, CRFs, and other records, and no names are used in any trial documentation to maintain confidentiality. All the records are being stored in locked cabinets. Clinical information will not be released without written permission, except when necessary for monitoring by trial monitors.

### Statistical analysis

The analyses have been described in detail in the full Statistical Analysis Plan. The main issues are summarized as follows.

Each intervention is hypothesized to be superior to the standard of care, and therefore the proposed analysis is intended to include all randomized participants. The primary analysis of mortality in children randomized to high-versus low-dose hydroxyurea will use time-to-event methods (Kaplan-Meier, log-rank test, proportional hazards models), stratified by center and other stratification factors (including the other randomizations and adjusting for initial hydroxyurea dose). If the primary superiority hypothesis is not met (i.e., high dose is not shown to be superior to low dose in terms of mortality), we will conduct secondary exploratory non-inferiority analyses to assess whether mortality is non-inferior with low dose compared to high-dose hydroxyurea. This will be conducted at a one-sided significance level (as we do not expect that low doses will be superior to high doses in terms of mortality). The planned non-inferiority margin is a 5% absolute difference in cumulative mortality at 3 years. Assuming no true difference between the arms, the expected mortality in the high-dose group will be 11% at 3 years; 1800 children will provide 91% power to demonstrate non-inferiority, assuming a 5% loss to follow-up (one-sided α=0.025). If the high-dose 3-year mortality is less than 7.5% (approximately 2.5/100 CY in Table 1), then the non-inferiority margin will be modified to a 3.5% absolute difference (without reference to the accumulating comparative data) to reflect the equivalent relative differences between arms.

Analyses of hospitalizations (malaria-specific and all-cause) will use Poisson regression if there is no evidence of over-dispersion, or negative binomial models if there is evidence of over-dispersion (p<0.01), using the number of hospitalizations as the count and the time at risk under follow-up as the exposure. Zero-inflated models will be used if there is statistical evidence (p<0.01) that more children than expected will not experience the outcome. Similar methods will be used to analyze other time-to-event and count outcomes.

Pre-specified subgroup analyses will include each of the other randomized allocations (i.e., exploration of interactions in the factorial design) together with the other randomization stratification factors (center, initial hydroxyurea dose). We will also investigate a priori whether there is any evidence for a different impact of the interventions according to age, hematological indices, and time in the trial (to assess for attenuation of effects over time). These are considered categorical variables, splitting at terciles, and as natural cubic splines.

Continuous secondary outcome measures will be analyzed using normal linear regression adjusted for baseline values and randomization stratification factors, with generalized estimating equations (independent working correlation) used to compare randomized groups across multiple time points. The frequency of hospital readmissions and adverse events will be tabulated by body system and randomized groups, and the number of events experienced by each participant will be compared across randomized groups using Fisher’s exact test.

In the within-trial analysis, the differential cost of treatment interventions will be related to their differential outcomes in terms of the primary outcome. The relative cost-effectiveness of alternative forms of management will then be assessed using standard decision rules, and a full stochastic analysis will be undertaken. A cost-utility analysis will also be conducted using a standard approach. The within-trial analysis will be augmented by extrapolation beyond the trial follow-up using decision-analytic modelling. The aim of this analysis will be to predict the implications of any differences in clinical endpoints in the trial for subsequent quality-adjusted survival duration and long-term resource costs. This will inform the question of whether any differences in drug costs between the treatment groups are offset by reductions in other treatment costs or health improvements in the long term.

### Ethical approval

Ethical approval was first obtained from the sponsor, Imperial College London, for a placebo-controlled trial of hydroxyurea under protocol V0.9 (dated 10/08/19), on 16/08/19 [Imperial College Research Ethics Committee (ICREC) number 19IC5453]. V0.9 was subsequently approved by the Mbale Regional Referral Hospital Research and Ethics Committee (MRRH-REC), on 21 ^st^ February 2020 (MRRH-OUT 032/20). Various issues then led to delays in beginning the trial, including COVID19, and the publication of data from other trials that resulted in the re-design of H-PRIME into an open-label high- versus low-dose trial instead of the original placebo-controlled design. Several protocol versions were approved during the subsequent period, none of which was implemented. The trial was finally activated under protocol V4.0 (dated 20/10/22), which was approved by MRRH-REC (MREC-OUT 032/20) on 4 ^th^ April, 2023, and by ICREC on 26 ^th^ May 2023. The trial was registered on ISRCTN (ISRCTN15724013) on 25/02/2020) and on the Pan Africa Clinical Trials PACTR (202401802272880) on 09/01/2024.

### Safety

The trial will be performed in participants who may benefit from the treatment. The principles of ICH GCP require both investigators and sponsors to follow specific procedures when notifying and reporting adverse events or reactions in clinical trials.

### Risks

Hydroxyurea, DHA-PQP, and CTX prophylaxis have been widely used in children with minimal risk, as have SP and penicillin V. However, all medications confer potential additional risks from their administration, and the trial will directly evaluate whether these potential risks are outweighed by improved survival and reduced hospitalization. Blood samples are being obtained from all children in the trial. However, the volumes of blood required have been minimized as much as possible and kept well within the maximum locally agreed volume.

### Benefits

Additional clinical personnel, regular clinical assessment of participants, and basic equipment for patient monitoring are available during the trial so that if SCD complications arise, they will be detected and treated more often. Pre-trial training included sign recognition of these complications and treatment training. Both are covered in detail in the trial Manual of Procedures (MOP.

### For the child

The direct benefits to the child and/or family (outlined in the patient information sheet (PIS)) include the following.

- Closer observation during the trial, which, as a result, allows doctors and nurses to make important changes to the child’s management.
- The dependable supply of standard of care medicines that could otherwise be subject to stockouts free of charge to parents.
- Additional education about their condition.

### For the centres

The direct benefits to the participating centres include:

- Support, capacity development, and training in the management of SCD in childhood and use of hydroxyurea therapy.
- Establishing or further developing SCD clinics.

### For health personnel

The direct benefits to health personnel are mainly the professional development of the members of the trial teams and clinical teams for the purposes of running the trial, including training in clinical trials, good clinical and laboratory practice, and research ethics. However, as mentioned above, they will also receive standardized training in the identification and treatment of SCD and relevant adverse events.

### Definitions

The definitions of EU Directive 2001/20/EC Article 2, based on the principles of ICH GCP, apply to this trial protocol. The definitions are given below.

### Medicinal products

An investigational medicinal product is defined as the tested investigational medicinal product (IMP) and comparators used in the trial (EU guidance ENTR/CT April 3, 2006, revision). H-PRIME included hydroxyurea, SP, DHA-PQP, CTX, and penicillin V.

Adverse reactions include unintended or unintended responses to drugs. Reactions to an IMP or comparator should be reported appropriately.

### Adverse Events

Adverse Events (AEs) include:

- An exacerbation of a pre-existing illness.
- An increase in the frequency or intensity of a pre-existing episodic event or condition.
- A condition (even though it may have been present prior to the start of the trial) that was detected after the trial drug administration.
- Continuous persistent disease or symptoms present at baseline worsen following administration of the trial treatment.

Adverse Events do not include:

- In medical or surgical procedures, the condition that leads to the procedure is an AE.
- Pre-existing disease or a condition present before treatment that does not worsen.
- Hospitalizations where no unintended or unintended response has occurred, such as elective cosmetic surgery or social admission.
- Overdose of medication without signs or symptoms

### Other notable events

Pregnancy-related events will not constitute SAEs unless they result in a condition that meets the seriousness criteria defining SAEs (e.g., septic abortion and congenital abnormality). However, these are notifiable events and, therefore, should be reported as soon as possible. No other notable events have occurred.

## Investigator responsibilities

### Investigator assessment

***Seriousness*.** When an AE occurs, the investigator responsible for the care of the participant must first assess whether the event is serious, using the definition given in Table 8.

**Table 8.** Adverse event definitions.

T erm	D efinition
Adverse Event (AE)	Any untoward medical occurrence in a patient or clinical trial subject to whom a medicinal product has been administered including occurrences that are not necessarily caused by or related to that product.
Adverse Reaction (AR)	Any untoward and unintended response to an investigational medicinal product related to any dose administered.
Unexpected Adverse Reaction (UAR)	An adverse reaction, the nature or severity of which is not consistent with the information about the medicinal product in question set out in the Summary of Product Characteristics (SPC) or Investigator Brochure (IB) for that product.
Serious Adverse Event (SAE) or Serious Adverse Reaction (SAR) or Suspected Unexpected Serious Adverse Reaction (SUSAR)	Respectively any adverse event, adverse reaction or unexpected adverse reaction that: ▪ Results in death ▪ Is life-threatening * ▪ Requires hospitalization or prolongation of existing hospitalization ** ▪ Results in persistent or significant disability or incapacity ▪ Consists of a congenital anomaly or birth defect ▪ Is another important medical condition ***

*The term life-threatening in the definition of a serious event refers to an event in which the patient is at risk of death at the time of the event; it does not refer to an event that hypothetically might cause death if it was more severe, for example, a silent myocardial infarction. **Hospitalisation is defined as an inpatient admission, regardless of length of stay, even if the hospitalisation is a precautionary measure for continued observation. Hospitalizations for a pre-existing condition that has not worsened or for an elective procedure do not constitute SAE. *** Medical judgement should be exercised in deciding whether an AE or AR is serious in other situations. The following should also be considered serious: important AEs or ARs that are not immediately life-threatening or do not result in death or hospitalization but may jeopardize the subject or may require intervention to prevent one of the other outcomes listed in the definition above, for example, a secondary malignancy, an allergic bronchospasm requiring intensive emergency treatment, seizures, or blood dyscrasias that do not result in hospitalization or development of drug dependency. Note: Suspected Unexpected Serious Adverse Reactions (SUSAR) will not be assessed in H-PRIME, as it falls outside the scope of the European Union Clinical Trial Regulations.

***Severity or grading of Adverse Events*.** The severity of AEs and/or ARs (serious and non-serious) in this trial should be graded using the Common Terminology Criteria for Adverse Events (CTCAE) v5.0 ^77^, with the exception of specific laboratory abnormalities listed in Table 4, which are graded as in the REACH trial, recognizing the lower normal levels in African populations and/or in individuals with SCD.

### Causality

The investigator responsible for the care of the participant must assess the causality of all serious events or reactions in relation to the trial medications, using the definitions in Table 9. Investigators should use clinical judgment, considering the chronological relationship between drug administration and the occurrence of adverse reactions, along with consensus knowledge about SCD in determining event causality. There are six categories: unrelated, unlikely, possible, probable, definitely related, and not assessable. These follow the WHO-Uppsala Monitoring Center criteria ^78^ but combine the unclassified and unclassifiable categories into one “not assessable” category and include an unrelated category because many of the drugs used in H-PRIME have known side effects based on many years of use. As all children are on multiple drugs, in some situations, it will be clear that a reaction is to one drug being taken and not to others being taken. These categories have been widely used in trials conducted across the EU and in previous trials of acutely sick children (including those with HIV infection) across Africa. If the causality assessment is unrelated or unlikely to be related, the event is classified as SAE or AE. If causality is assessed as possible, probable, or definitely related, then the event is classified as SAR or AR. If any doubt about causality exists, the local investigator should inform the trial coordination center who will notify the Chief Investigators. In some cases, clinicians may be asked to advise them.

**Table 9.** Causality assessment.

Relationship	WHO	Description *
Unrelated **	-	There is no evidence of any causal relationship and there is another probable/ definite explanation for the event (e.g. the participant’s clinical condition, other concomitant treatment)
**Unlikely**	Unlikely	There is little evidence to suggest there is a causal relationship (e.g. the event did not occur within a reasonable time after administration of the trial medication). There is another reasonable explanation for the event (e.g. the participant’s clinical condition, other concomitant treatment).
**Possible**	Possible	There is some evidence to suggest a causal relationship (e.g. because the event occurs within a reasonable time after administration of the trial medication). However, the influence of other factors may have contributed to the event (e.g. the participant’s clinical condition, other concomitant treatments).
**Probable**	Probable	There is evidence to suggest a causal relationship and the influence of other factors is unlikely.
**Definitely**	Certain	There is clear evidence to suggest a causal relationship and other possible contributing factors can be ruled out.
**Not ** assessable ***	Unassessable Unclassifiable Conditional Unclassified	There is insufficient or incomplete evidence to make a clinical judgement of the causal relationship.

* All points should be compliant ** An adverse event is classified as “Unrelated” when there is no plausible time relationship to drug intake and the event can be fully explained by other factors such as underlying disease or other drugs. This will include coincidental events. *** The term includes Unassessable / Unclassifiable / Conditional / Unclassified events where additional data is required, or data cannot be verified.

### Reporting procedures

During each clinical review, the clinician or nurse checks for AEs. In the event of an abnormal clinical or laboratory finding by the trial clinician, children receive appropriate treatment according to the national or WHO clinical guidelines, including admission for assessment and/or treatment where appropriate. All non-serious AEs and ARs, whether expected or not, are being recorded in the child’s medical notes and, if appropriate, reported in the clinical symptoms section of the appropriate CRF, and data entered within the agreed timescale. As medications used in the trial are all licensed in children, clinical or laboratory AEs are reported on CRFs only if they are grade 3 or 4, serious or if they lead to modification, or temporary or permanent discontinuation of any trial treatment. SAEs and SARs are notified to the trial coordinating center at the MCRI and the Chief Investigator within one working day of the trial team becoming aware of the event. The Trial coordinating center ensures that the Mbale Regional Referral Hospital Research Ethics Committee and the Uganda National Drug Authority are informed within seven days of the investigator becoming aware of the event. The Uganda National Council for Science and Technology is being informed of this through regular reports.

The reporting procedures are detailed in the safety reporting SOP. Any questions concerning adverse event reporting are being directed to the trial coordination center in the first instance via email or telephone. The SAEs are reviewed immediately by a designated clinician (SAE reviewer). The causality assessment given by the local investigator at the hospital cannot be overruled; in the case of disagreement, both opinions are provided in subsequent reports.

SAEs are being reported to the trial coordination center using an SAE CRF, which is being completed, scanned, and sent electronically to the H-PRIME trial coordination center at the MCRI within one working day. The SAE form asks about the nature of the event, date of onset, severity, corrective therapies given, outcome, and causality (i.e., unrelated, unlikely, possible, probably, definitely). The investigator signs the CRF. Additional information is being sent within 5 days in cases where the event had not already resolved at the time of reporting.

Local investigators are reporting all SAEs, as required by their Local Research Ethics Committee. Any questions concerning adverse event reporting are being directed to the Chief Investigator in the first instance.

### Public engagement

Community engagement is occuring through regular meetings with local communities involving hospital community representatives. Information and feedback are being provided and received at these meetings. Each site either uses their existing Community Advisory Board (CAB) or has formed a specific patient liaison group to feedback concerns and questions from the community and hear about the latest developments in the trial and the wider scientific community, where possible. Dialogue with these groups will be maintained through regular briefing meetings during the course of the trial, and is a standing item at each TSC meeting. The results of this trial will be disseminated locally through community and national meetings with a wider healthcare professional community.

### National policymakers

The site’s principal investigators will discuss ongoing trials with the Ugandan Ministry of Health. A summary or brief summary will be produced to highlight the trial results and the next steps required to inform rational evidence-based guidelines.

### International policymakers

We will engage with international policymakers, including the World Health Organization, at the conclusion of the trial.

## Discussion

H-PRIME is a large phase III multicentre factorial randomized trial investigating the pragmatic management of children with SCD in a real-world setting in Eastern Uganda. Such trials have the potential to lead to policy-changing recommendations for the treatment of this important, but neglected disease. Factorial designs are a highly efficient approach to answering multiple relevant clinical questions.

Recruitment began on January 16, 2024, across four clinical centers in Eastern Uganda: Mbale, Soroti, Atutur, and Ngora. As of February 17, 2025, the trial enrolled 1,487 of the targeted 1,800 children aged 1–10 years, with full enrolment expected to be completed by mid-2025.

Participants are currently receiving assigned interventions and follow-ups, with active data collection and safety monitoring. In addition, dedicated substudies, including pharmacokinetic (PK) and cardiac safety assessments, are underway at the Mbale site.

The trial has obtained full ethical approval (details provided in the main protocol document) and is registered with the International Standard Randomized Controlled Trial Number (ISRCTN15724013) and the Pan African Clinical Trials Registry (PACTR202401802272880). Both registrations are active and current.

Interim safety monitoring is being conducted in accordance with protocol-defined procedures. Grade 4 adverse events, such as critical blood count abnormalities, are flagged for clinical action and oversight is provided through independent monitoring and data safety review structures.

The trial is being conducted by a collaborative international consortium that includes the Mbale Clinical Research Institute (Uganda), Medical Research Council Clinical Trials Unit at UCL (UK), Imperial College London (UK), KEMRI-Wellcome Trust (Kenya), and Cincinnati Children’s Hospital (USA).

### Protocol version changes

Various versions of the protocol were approved, but not implemented, between 2019 and 2022 because of delays pertaining to COVID19 and then subsequent developments in the field. The trial was first activate under protocol V4.0 (dated 20/10/22), which was approved by MRRH-REC on 4 ^th^ April, 2023, and by ICREC on 26 ^th^ May 2023. A minor revision to Version 4.0.1 (18/09/23) was approved by MRRH-REC on 28/09/23 and by ICREC on 22/11/23. The current version of the protocol, V5.0 (18/12/24), which included no changes to the trial randomisation or outcome measures but included a number of important clarifications, changes to the data collection materials, and additional statistical analysis plans, was approved by MRRH-REC on 14/03/25 and by ICREC on 23/04/25.

## Data Availability

No data are associated with this article. The ownership of the H-PRIME dataset lies with the H-PRIME TSC, which will approve all requests for the use of trial data before and after the trial ends, based on a controlled access approach (requests before the end of the trial also to be approved by the H-PRIME DMC). No data that compromise the confidentiality of the research participants or their communities will be shared. No collaborating research partner will transfer data to any third party without written consent from the other partners. On completion of the trial, local researchers will have unrestricted access rights to datasets collected through this collaborative research project. We will follow the funder’s regulations regarding to Intellectual Property (IP). As this relates to legal issues, IP issues are laid out in contracts between partners. The H-PRIME dataset will be held electronically for at least 20 years after the end of the trial in accordance with MRC policy. As mentioned above, proposals to use H-PRIME data and samples will be welcomed and supported widely, where this does not conflict with existing plans within the trial team (e.g., as described in the primary and secondary objectives of the trial above). Researchers wishing to access data should first contact the Trial Management Group. The patient information sheets, consent forms, trial protocol, and case report forms can be found as Extended Data on Harvard Dataverse repository under the title: “Extended Data for: Hydroxyurea - Pragmatic Reduction In Mortality and Economic burden (H-PRIME): A 2x2x2 factorial randomised open-label trial investigating practical approaches to the treatment of sickle cell disease at four sites in Eastern Uganda: ISRCTN 15724013” (DOI: https://doi.org/10.7910/DVN/DKR3LX) ^79^. The deposited file consists of the following: 1_HPRIME_Form1_screening_v3.0_20241218_clean.docx (form used for screening children for eligibility) 2_HPRIME_Form2_eligibility_v3.0__20241218_clean.docx (pre-enrollment eligibility form) 3_HPRIME_Form3_baseline_v3.0__20241218_clean.docx (form used to collect baseline data) 4_HPRIME_Form4_randomisation_v3.0__20241218_clean.docx (form used to randomize children) 5_HPRIME_Form5_medication_v3.0__20241218_clean (form used to prescribe trial drugs) 6_HPRIME_Form6_follow-up_v3.0_20241218_clean.docx (data collection form for routine follow-ups) 7_HPRIME_Form7_grade 3or4 AE_v3.0_20241218_clean.docx (form used to collect data on grade 3 or 4 adverse events) 8_HPRIME_Form8_SAE_v3.0_20241218_clean.docx (form used to collect data on severe adverse events) 9A_HPRIME_Form9A_research haematology_v3.0_20241218_clean.docx (form used to collect data on research haematology full blood count data) 9B_HPRIME_Form9B_research haematology_v3.0_20241218_clean.docx (form used to collect data on research haematology haemoglobin electrophoresis and HPLC data) 10_HPRIME_Form10_local FBC_v3.0_20241218_clean.docx (form used to capture haematological data from samples taken for patient management purposes) 11A_HPRIME_Form11A_research renal and liver function_v3.0_20241218_clean.docx (form used to capture data from research renal function tests) 11B_HPRIME_Form11B_local renal and liver function_v3.0_20241218_clean.docx (form used to capture data from research liver function tests) 12_HPRIME_Form12_detailed adherence_v3.0_20241218_clean.docx (form used to capture data on drug adherence) 13_HPRIME_Form13_PedsQL_v3.0_20241218_clean.docx (form used to capture data on quality of life) 14A_HPRIME_Form14A_blood slide v3.0_20241218_clean.docx (form used to capture data on the results of malaria blood film tests) 14B_HPRIME_Form14B_blood culture v3.0_20241218_clean.docx (form used to accompany blood culture tests) 15_HPRIME_Form15_LTFU and withdrawal v3.0_20241218_clean.docx (form used to document loss to follow-up or trial withdrawal) 16_HPRIME_Form16_acutely sick visit_v3.0_20241218_clean.docx (form used to collect data during unscheduled study visits) 17_HPRIME_Form 18a_DHA/PQP levels v3.0_20241218_clean.docx (form used to accompany samples for DHA/PQP measurements) 18_HPRIME_Form18_Cardiac Study v3.0_20241218_clean.docx (for used to collect data for the cardiac sub study) 19_HPRIME_Form19_withdrawal of consent v3.0__20241218_clean.docx (form used to document withdrawal of consent to the trial) 20_grade 4 laboratory events v3.0_20241218_clean.docx (form used to feedback results of grade 4 laboratory events) 101_H-PRIME patient information sheet – parents and carers v2.06.docx (patient information sheet for parents and carers) 102_H-PRIME TRIAL patient information sheet older children v2.06.docx (patient information sheet for older children) 103_H-PRIME consent form for screening v2.06.docx (form used to document consent for screening) 104_H-PRIME TRIAL consent form - parents and carers v2.06.docx (form used to record consent from parents or guardians) 105_H-PRIME TRIAL assent form v2.06.docx (form used to record assent from trial participants) 106_H-PRIME TRIAL patient information sheet specimen storage v2.06.docx (patient information sheet to describe specimen storage) 107_H-PRIME TRIAL consent form specimen storage v2.06.docx (consent form for specimen storage) Data are available under the terms of the Creative Commons Attribution 4.0 International license (CC-BY 4.0). All data will be made available under the terms of the Creative Commons Zero "No rights reserved" data waiver (CC0 1.0 Public domain dedication). The SPIRIT checklist and flow diagram for the trial is published under a CC BY 4.0 licence on Harvard Dataverse under the title “SPIRIT 2013 Checklist: Hydroxyurea - Pragmatic Reduction In Mortality and Economic burden (H-PRIME): A 2x2x2 factorial randomised open-label trial investigating practical approaches to the treatment of sickle cell disease at four sites in Eastern Uganda: ISRCTN 15724013” DOI https://doi.org/10.7910/DVN/QUGG6P ^78^." The reference ^78^ Data are available under the terms of the Creative Commons Attribution 4.0 International license (CC-BY 4.0).
