# Supplementary material for: Hydroxyurea - Pragmatic Reduction In Mortality and Economic burden (H-PRIME): A 2x2x2 factorial randomised open-label trial investigating practical approaches to the treatment of sickle cell disease at four sites in Eastern Uganda
Source: Wellcome Open Res. 2025 May 16;10:244. [Version 1] doi: 10.12688/wellcomeopenres.24096.1 (PMC13620357; doi:10.12688/wellcomeopenres.24096.1)
Supplement: Peer review history

# Reviewer report: Reviewer response for version 1

**DOI:** 10.21956/wellcomeopenres.26583.r147925

**Published:** 2026-04-01

**Authors:** Amza Ali, Monika Parshad-Asnani

**Affiliations:** 1 The University of the West Indies, Kingston, St. Andrew Parish, Jamaica

**Hydroxyurea in SCD protocol**

**H-PRIME Trial Protocol**

 The protocol by Olupot-Olupot and colleagues describes the Hydroxyurea – Pragmatic Reduction in Mortality and Economic Burden (H-PRIME) trial, a multicentre 2×2×2 factorial randomized trial evaluating pragmatic treatment strategies for children with sickle cell disease (SCD) in Eastern Uganda. The investigators aim to determine whether simplified (low and high dose) hydroxyurea dosing, enhanced malaria prophylaxis, and enhanced lifelong antibacterial prophylaxis can reduce mortality, all-cause hospitalization, and economic burden among children living with SCD.

 This study addresses a clinically important and policy‑relevant question. SCD remains a major contributor to childhood morbidity and mortality in sub‑Saharan Africa, yet much of the evidence guiding management derives from studies conducted in high‑income settings. The pragmatic orientation of this trial, particularly its focus on simplified treatment strategies that may be feasible in routine clinical environments, is therefore a notable strength. The factorial design further enhances efficiency by allowing several clinically relevant hypotheses to be evaluated simultaneously.

 Overall, the protocol is carefully conceived and addresses a major evidence gap. The following comments are offered with the aim of strengthening methodological clarity and potential policy impact of the study.

**Major Comments**

 1. Assumptions underlying the power calculations

 The protocol presents power calculations based on assumed mortality and hospitalization rates among children receiving low‑dose hydroxyurea. While these assumptions appear reasonable, it would be helpful if the investigators cla

 rified the basis for these estimates and explicitly cited the studies informing them.

 In addition, the assumption of approximately 5% loss to follow‑up over a four‑year follow‑up period may be optimistic for a longitudinal paediatric trial conducted in a resource‑limited setting, unless this has been the regular clinical experience. Attrition related to geographic mobility, socioeconomic pressures, and competing health priorities is often higher in comparable settings. Sensitivity analyses exploring the effect of higher attrition rates on statistical power would strengthen the justification for the proposed sample size.

 2. Factorial design and potential interaction effects

 The 2×2×2 factorial design represents an efficient and appropriate approach to evaluating multiple pragmatic interventions simultaneously. However, the analysis plan appears to assume minimal interaction between the hydroxyurea dosing strategy and the malaria and antimicrobial prophylaxis interventions.

 Given emerging evidence that hydroxyurea may influence infection risk, inflammatory pathways, and clinical events in SCD and consequently hospitalization risk, it would be helpful for the investigators to clarify whether interaction effects are anticipated and how such interactions will be evaluated statistically. Clarification regarding potential interaction effects between hydroxyurea dosing and the malaria and antimicrobial prophylaxis (considered below) interventions would be helpful. Also, the authors should consider other possible interactions across treatment arms eg antibiotics with possible antimalarial effects. Perhaps the statistical tools and expertise will be able to resolve this adequately as this will be very important in understanding the clinical impact of each intervention and to have confidence in future policy making.

 3. Antibacterial prophylaxis

 Once monthly long-active penicillin has been successfully used in other settings. It would be helpful to have clarity as to why this economical and reliable alternative was not used in this study.

 4. Pragmatic monitoring strategy for hydroxyurea

 The trial adopts a pragmatic monitoring approach in which laboratory results are not routinely returned to clinicians except in cases of severe abnormality. This design reflects the realities of many health systems in sub‑Saharan Africa and represents an innovative attempt to evaluate scalable treatment strategies.

 Nevertheless, additional clarification would be useful regarding the schedule of research laboratory testing, how abnormal laboratory findings will be communicated to treating clinicians, and the predefined criteria for treatment interruption or discontinuation in cases of suspected toxicity.  This would help reassure readers that patient safety remains appropriately safeguarded.

 5. Neurological complications and long‑term morbidity

 The protocol appropriately emphasizes infectious complications as important drivers of mortality among children with SCD in sub‑Saharan Africa. However, neurological complications represent a substantial and often under‑recognised component of the disease burden. Stroke, silent cerebral infarction, epilepsy, neurocognitive impairment, and neuropathic pain all contribute significantly to long‑term disability and economic burden in affected individuals.

 Stroke and silent cerebral infarction are among the most common neurological complications of childhood SCD and are strongly associated with cognitive impairment and educational disadvantage ^1^. It would be helpful to have clarity on whether the patients enrolled had transcranial doppler (TCD) monitoring at baseline since it is known that TCD measurements are strongly recommended in this age group as part of screening.  Seizure disorders may occur in association with overt or silent cerebrovascular injury and have been documented in longitudinal cohorts of patients with SCD ^2,3^. In addition, neuropathic pain has increasingly been recognised as an important contributor to chronic pain syndromes and reduced quality of life in SCD ^4^ . Several workers further highlight the complexity of managing pain in this population ^5^.

 More recently, broader neurological manifestations of SCD, including cognitive impairment and chronic neurological morbidity, have been emphasized as key determinants of long‑term outcomes. While the introduction briefly acknowledges cerebrovascular injury, the protocol does not clearly indicate whether neurological outcomes will be systematically recorded.

 Given the stated aim of evaluating mortality, morbidity, and economic burden, consideration could be given to capturing neurological events—such as stroke, seizures, or persistent neurological deficits—as important secondary outcomes. Even clinical reporting of such events could substantially enrich interpretation of the trial findings.

 6. Health economic evaluation

 The inclusion of an economic evaluation is an important strength of the protocol, in particular the focus on cost-effectiveness of the three interventions However, the analytic perspective adopted for the economic analysis is not entirely clear.

 The economic burden associated with SCD in low‑ and middle‑income settings frequently extends beyond direct healthcare costs and includes caregiver productivity losses, transportation costs for clinic attendance, school absenteeism, and long‑term disability related to chronic neurological complications. Clarification regarding whether the economic evaluation will adopt a health‑system perspective, a societal perspective, or both would improve interpretability and policy relevance. Extrapolations of future earnings loss from chronic disabilities, especially neurocognitive ones would be valuable. With mortality as the primary outcome, it would be helpful to have clarity about how the authors will determine cost-effectiveness based on lives saved.

 Finally, it would be helpful to have further insight into how quality‑of‑life data will be incorporated into the economic evaluation.

 7. Quality‑of‑life outcomes

 The protocol identifies quality of life as an outcome but as a secondary or ancillary measure. Given the chronic nature of SCD and its substantial impact on physical, social, and educational functioning, health‑related quality of life represents an important outcome in paediatric populations.

 Clarification would be helpful regarding which quality‑of‑life instrument will be used for analysis and whether disease‑specific measures will be considered. Two generic quality-of-life instruments (EQ-5D-Y and the PedsQL Generic Core Scale) are referenced in the protocol; however, it is not entirely clear which instrument will be used for the primary quality-of-life analyses and which will inform the economic evaluation.

 Minor Comments

 1. The protocol mentions monitoring for potential cardiotoxicity associated with dihydroartemisinin‑piperaquine. Clarification would be helpful regarding how this will be done ie clinically or with periodic scheduled EKGs?

 2.The criteria for child assent could be more clearly defined. Clarification would be helpful regarding how individual assent and withdrawal will be handled when multiple children from the same family are enrolled, particularly where one child declines participation either at the time of enrollment or during the study.

 Overall Assessment

 This protocol describes a well‑conceived and highly relevant pragmatic clinical trial addressing important evidence gaps in the management of sickle cell disease in sub‑Saharan Africa. The large sample size, implementation strategy, and factorial design represent notable strengths that enhance its potential policy relevance.

 Clarification of some methodological aspects such as assumptions underlying the power calculations, monitoring procedures for hydroxyurea therapy, and the scope of the economic evaluation, would further strengthen the protocol. The H‑PRIME trial has the potential to generate valuable evidence to inform pragmatic and scalable strategies for improving outcomes among children living with sickle cell disease in resource‑limited settings.

 References

 1. DeBaun MR, Kirkham FJ. Central nervous system complications and management in sickle cell disease. *Blood*. 2016;127(7):829-838. doi:10.1182/blood-2015-09-618579

 2. Nawaiseh MB, Yassin AM, Al-Sabbagh MQ, et al. Abnormal Neurologic Findings in Patients With Sickle Cell Disease Without a History of Major Neurologic Events. *Neurol Clin Pract*. Feb 2024;14(1):e200215. doi:10.1212/cpj.0000000000200215

 3. Liu JE, Gzesh DJ, Ballas SK. The spectrum of epilepsy in sickle cell anemia. *Journal of the neurological sciences*. May 1994;123(1-2):6-10. doi:10.1016/0022-510x(94)90196-1

 4. Glaros A, Brandow AM. Neuropathic pain in sickle cell disease: measurement and management. *Hematology Am Soc Hematol Educ Program*. Dec 4 2020;2020(1):553-561. doi:10.1182/hematology.2020000142

 5. Tran H, Gupta M, Gupta K. Targeting novel mechanisms of pain in sickle cell disease. *Blood*. Nov 30 2017;130(22):2377-2385. doi:10.1182/blood-2017-05-782003

Is the study design appropriate for the research question?

Yes

Is the rationale for, and objectives of, the study clearly described?

Yes

Are sufficient details of the methods provided to allow replication by others?

Partly

Are the datasets clearly presented in a useable and accessible format?

Not applicable

Reviewer Expertise:

1. Amza Ali - Neurology; Sickle Cell neurological disease; Health economics. 2. Monika Parshad-Asnani - Sickle Cell disease and Quality of Life

We confirm that we have read this submission and believe that we have an appropriate level of expertise to confirm that it is of an acceptable scientific standard.

## References

[1] : Central nervous system complications and management in sickle cell disease. *Blood* .2016;127(7) : 10.1182/blood-2015-09-618579829-838 10.1182/blood-2015-09-618579 26758917

[2] : Abnormal Neurologic Findings in Patients With Sickle Cell Disease Without a History of Major Neurologic Events. *Neurology Clinical Practice* .2024;14(1) : 10.1212/CPJ.0000000000200215 10.1212/CPJ.0000000000200215 PMC1075909138173541

[3] : The spectrum of epilepsy in sickle cell anemia. *Journal of the Neurological Sciences* .1994;123(1-2) : 10.1016/0022-510X(94)90196-16-10 10.1016/0022-510X(94)90196-1 8064323

[4] : Targeting novel mechanisms of pain in sickle cell disease. *Blood* .2017;130(22) : 10.1182/blood-2017-05-7820032377-2385 10.1182/blood-2017-05-782003 PMC570978629187376

# Response

**Published:** 2026-07-09

**Authors:** Thomas N. Williams

**Affiliations:** KEMRI, Kenya

**Reviewer 2:** **Major Comments**

**Q1**. Assumptions underlying the power calculations

 The protocol presents power calculations based on assumed mortality and hospitalization rates among children receiving low‑dose hydroxyurea. While these assumptions appear reasonable, it would be helpful if the investigators clarified the basis for these estimates and explicitly cited the studies informing them.

 In addition, the assumption of approximately 5% loss to follow‑up over a four‑year follow‑up period may be optimistic for a longitudinal paediatric trial conducted in a resource‑limited setting, unless this has been the regular clinical experience. Attrition related to geographic mobility, socioeconomic pressures, and competing health priorities is often higher in comparable settings. Sensitivity analyses exploring the effect of higher attrition rates on statistical power would strengthen the justification for the proposed sample size.

**R1.** The studies on which the mortality and hospitalisation rates of children on low-dose hydroxyurea are already cited in the sample size section on power “Data from a range of recent relevant trials ^18^^, ^^29^ suggest that a range of mortality rates is plausible among children treated with low-dose hydroxyurea in Africa.” For greater clarity, we have moved the citations to the end of the sentence. The reviewers make the same point as reviewer 1 about attrition. Please see R1 above.

**Q2.** Factorial design and potential interaction effects

 The 2×2×2 factorial design represents an efficient and appropriate approach to evaluating multiple pragmatic interventions simultaneously. However, the analysis plan appears to assume minimal interaction between the hydroxyurea dosing strategy and the malaria and antimicrobial prophylaxis interventions.

 Given emerging evidence that hydroxyurea may influence infection risk, inflammatory pathways, and clinical events in SCD and consequently hospitalization risk, it would be helpful for the investigators to clarify whether interaction effects are anticipated and how such interactions will be evaluated statistically. Clarification regarding potential interaction effects between hydroxyurea dosing and the malaria and antimicrobial prophylaxis (considered below) interventions would be helpful. Also, the authors should consider other possible interactions across treatment arms eg antibiotics with possible antimalarial effects. Perhaps the statistical tools and expertise will be able to resolve this adequately as this will be very important in understanding the clinical impact of each intervention and to have confidence in future policy making.

**R2.** The efficiency of the factorial design depends on the assumption of no/minimal interactions, so yes, this is assumed in the analysis. For each randomisation, subgroup analyses are pre-specified by the other two randomisations so this assumption will be checked for all randomisations, using standard heterogeneity tests. We have added text on this point in the section on sample size.

**Q3.** Antibacterial prophylaxis

 Once monthly long-active penicillin has been successfully used in other settings. It would be helpful to have clarity as to why this economical and reliable alternative was not used in this study.

**R3.** While we agree that long-acting penicillins are a simple and useful intervention these are not widely and reliably available in Uganda and are not mentioned in the Uganda Guidelines for the management of children with SCA which stipulate Penicillin V. We hope this clarifies this question. We’ve not amended the protocol accordingly.

**Q4.** Pragmatic monitoring strategy for hydroxyurea

 The trial adopts a pragmatic monitoring approach in which laboratory results are not routinely returned to clinicians except in cases of severe abnormality. This design reflects the realities of many health systems in sub‑Saharan Africa and represents an innovative attempt to evaluate scalable treatment strategies.

 Nevertheless, additional clarification would be useful regarding the schedule of research laboratory testing, how abnormal laboratory findings will be communicated to treating clinicians, and the predefined criteria for treatment interruption or discontinuation in cases of suspected toxicity.  This would help reassure readers that patient safety remains appropriately safeguarded.

**R4. **Thank you for making this important point. The time points for the various assessments are already listed in the section on clinical follow-up but to make it easier to follow we have now included the full trial schedule as an Appendix. We already state the criteria for feeding back results in the section on trial treatment on Page 12 as “…. if severely abnormal values are detected, as defined by grade 4 laboratory adverse events (AEs), as outlined in Table 4.” We have modified the following sentence to clarify the procedure as follows “Such results are being communicated to site clinicians by telephone as soon as they are identified and such calls are recorded in a log held centrally at the trial laboratory for monitoring purposes.” We hope this addresses the reviewers’ concerns.

**Q5.** Neurological complications and long‑term morbidity

 The protocol appropriately emphasizes infectious complications as important drivers of mortality among children with SCD in sub‑Saharan Africa. However, neurological complications represent a substantial and often under‑recognized component of the disease burden. Stroke, silent cerebral infarction, epilepsy, neurocognitive impairment, and neuropathic pain all contribute significantly to long‑term disability and economic burden in affected individuals.

 Stroke and silent cerebral infarction are among the most common neurological complications of childhood SCD and are strongly associated with cognitive impairment and educational disadvantage. It would be helpful to have clarity on whether the patients enrolled had transcranial doppler (TCD) monitoring at baseline since it is known that TCD measurements are strongly recommended in this age group as part of screening.  Seizure disorders may occur in association with overt or silent cerebrovascular injury and have been documented in longitudinal cohorts of patients with SCD. In addition, neuropathic pain has increasingly been recognised as an important contributor to chronic pain syndromes and reduced quality of life in SCD. Several workers further highlight the complexity of managing pain in this population.

 More recently, broader neurological manifestations of SCD, including cognitive impairment and chronic neurological morbidity, have been emphasized as key determinants of long‑term outcomes. While the introduction briefly acknowledges cerebrovascular injury, the protocol does not clearly indicate whether neurological outcomes will be systematically recorded.

 Given the stated aim of evaluating mortality, morbidity, and economic burden, consideration could be given to capturing neurological events—such as stroke, seizures, or persistent neurological deficits—as important secondary outcomes. Even clinical reporting of such events could substantially enrich interpretation of the trial findings.

**R5. **Given that the trial is pragmatic and aimed at looking at the real-world situation, we are not conducting TCD measurements as part of H-PRIME. The method is time-consuming and few machines and technicians are available in Uganda. The inclusion of TCD measurements would have been costly and time consuming and would impair generalizability of our trial. An existing clinically defined stroke is an exclusion criterion. Thereafter, there is no systematic recording of neurological outcomes other than as Adverse Events. The impacts of neurological events detected through AE reporting will be reflected in the analysis through costs of healthcare utilization and impacts on quality of life. We have added a section justifying this approach and clarifying our approach to the capture neurological complications in the section on clinical assessment.

**Q6.** Health economic evaluation

 The inclusion of an economic evaluation is an important strength of the protocol, in particular the focus on cost-effectiveness of the three interventions However, the analytic perspective adopted for the economic analysis is not entirely clear.

 The economic burden associated with SCD in low‑ and middle‑income settings frequently extends beyond direct healthcare costs and includes caregiver productivity losses, transportation costs for clinic attendance, school absenteeism, and long‑term disability related to chronic neurological complications. Clarification regarding whether the economic evaluation will adopt a health‑system perspective, a societal perspective, or both would improve interpretability and policy relevance. Extrapolations of future earnings loss from chronic disabilities, especially neurocognitive ones would be valuable. With mortality as the primary outcome, it would be helpful to have clarity about how the authors will determine cost-effectiveness based on lives saved.

 Finally, it would be helpful to have further insight into how quality‑of‑life data will be incorporated into the economic evaluation.

**R6. **This is a similar point to that brought up by reviewer 1. Please see our response to reviewer 1.

**Q7.** Quality‑of‑life outcomes

 The protocol identifies quality of life as an outcome but as a secondary or ancillary measure. Given the chronic nature of SCD and its substantial impact on physical, social, and educational functioning, health‑related quality of life represents an important outcome in paediatric populations.

 Clarification would be helpful regarding which quality‑of‑life instrument will be used for analysis and whether disease‑specific measures will be considered. Two generic quality-of-life instruments (EQ-5D-Y and the PedsQL Generic Core Scale) are referenced in the protocol; however, it is not entirely clear which instrument will be used for the primary quality-of-life analyses, and which will inform the economic evaluation.

**R7. **This is a similar point to that brought up by reviewer 1. Please see our response to reviewer 1.​​​​​​​

**Minor Comments** **Q1.** The protocol mentions monitoring for potential cardiotoxicity associated with dihydroartemisinin‑piperaquine. Clarification would be helpful regarding how this will be done ie clinically or with periodic scheduled EKGs?

**R1. **This is a similar point to that brought up by reviewer 1. Please see our response to reviewer 1.

 The schedule of assessment is also now included in full as an appendix.

**Q2.** The criteria for child assent could be more clearly defined. Clarification would be helpful regarding how individual assent and withdrawal will be handled when multiple children from the same family are enrolled, particularly where one child declines participation either at the time of enrollment or during the study.

**R2. **This is a similar point to that brought up by reviewer 1. Please see our response to reviewer 1.

# Reviewer report: Reviewer response for version 1

**DOI:** 10.21956/wellcomeopenres.26583.r123846

**Published:** 2025-06-05

**Authors:** Arlene Smaldone

**Affiliations:** 1 Columbia University School of Nursing, New York, USA

Thank you for the opportunity to review this protocol. The study addresses an important topic as sickle cell disease is prevalent in sub Saharan Africa and treatments available to children in the United States and other developed countries are not routinely available to children in Africa.  This study, through use of a factorial design, proposes to test three important hypotheses which, if supported, could improve the lives of children living with sickle cell disease. My specific questions and recommendations to improve the protocol follow.

 1. Power analysis: The assumptions guiding the power analyses for each hypothesis is unclear - are these guided by the literature (if so, provide citations) or fully investigator driven.  The assumption for 5% loss to follow up over a 4-year period of time seems to be unrealistic and could compromise study results.

 2. Randomization - size of permuted blocks for randomization needs clarification. Further, while it is clearly stated that multiple children in a family would be assigned the same treatment, how this affects permuted block size and the overall randomization scheme is unclear and needs to be addressed.

 3. Language of study materials available to participants including consent, assent and scales such as quality of life scales is not stated and needs to be.

 4.  Consent - age or other criteria for child assent is not stated. Also, for study withdrawal only the caregiver is considered. Children also should have the ability to decide about study withdrawal.

 5. Quality of life assessment is mentioned as an overarching aim. Quality of life is a very important consideration for youth with sickle cell disease. Surprisingly, quality of life is relegated to “other outcomes” which diminished its importance in this trial. I’d encourage the investigators to reconsider this. Further, two different generic quality of life assessments are mentioned: EQ 5D Y and PedsQL generic core scale. Which scale will be used in analyses.  Will EQ SD Y be used for the cost effectiveness substudy and PedsQL for other analyses?  The protocol should rectify this.  Finally, I think the investigators miss an important opportunity to examine disease specific quality of life of children with sickle cell disease and would encourage them to add the PedsQL Sickle Cell module to their battery of instruments.

 6. Health economics sub study – the perspective to inform the proposed cost effectiveness analysis is at the healthcare provider level.  Far more important, in my opinion, is consideration of a societal perspective.  The investigators do consider time lost from school but should also consider caregiver costs such as time lost to work for child illness, medical appointments, etc.

 7. Clinical assessment #8 it is unclear how cardiac arrhythmias will be identified – via ECG monitoring or reliance on presence of symptoms only

Is the study design appropriate for the research question?

Yes

Is the rationale for, and objectives of, the study clearly described?

Yes

Are sufficient details of the methods provided to allow replication by others?

Partly

Are the datasets clearly presented in a useable and accessible format?

Not applicable

Reviewer Expertise:

Testing interventions to improve adherence and quality of life in youth with sickle cell disease

I confirm that I have read this submission and believe that I have an appropriate level of expertise to confirm that it is of an acceptable scientific standard, however I have significant reservations, as outlined above.

# Response

**Published:** 2025-06-23

**Authors:** Thomas N. Williams

**Affiliations:** KEMRI, Kenya

We thank Dr Smaldone for their comments and respond as follows: **Question 1. ** Power analysis: The assumptions guiding the power analyses for each hypothesis is unclear - are these guided by the literature (if so, provide citations) or fully investigator driven.  The assumption for 5% loss to follow up over a 4-year period of time seems to be unrealistic and could compromise study results. **Response 1.  ** Regarding our power calculations for mortality, we state on page 8 that “Data from a range of recent relevant trials (18,29) suggest that a range of mortality rates is plausible among children treated with low-dose hydroxyurea in Africa”. The references to two papers by Tshilolo and Abdullahi are the basis for the figures displayed in Table 1. **For clarity, we will add these references to the footnote to Table 1 at revision stage.**   Regarding the rates of hospital admission for malaria, the pre-intervention rate was derived from Macharia et al, 2017, while the figure for all-cause hospital admission, was based on data from Uyoga et al, 2019. Both studies were conducted in an area with lower malaria endemicity than Uganda and therefore probably underestimate the true power of the trial for these events. **We will add these references to the power calculation section of our paper and clarify these assumptions at revision stage. **   The assumption of 5% loss to follow up over 4 years is based on our experience in the REACH trial and in numerous previous trials that we have conducted in Eastern Uganda. We do not consider the assumption to be unrealistic. **We will also add text to the paper to defend this assumption at revision stage.** **Question 2. ** Randomization - size of permuted blocks for randomization needs clarification. Further, while it is clearly stated that multiple children in a family would be assigned the same treatment, how this affects permuted block size and the overall randomization scheme is unclear and needs to be addressed. **Response 2.  ** Two different sizes of permuted blocks were used but were we are not making these sizes public at this stage for reasons of allocation concealment. ‘Family randomisation’ was treated as a separate stratum within the weight band stratification so there was one randomisation for each family. **We will clarify these points** **at revision stage** **.** **Question 3. ** Language of study materials available to participants including consent, assent and scales such as quality of life scales is not stated and needs to be. **Response 3.  ** All these materials are available in both English and all 5 local languages - Ateso, Kumam, Lugwere, Luganda, and Lumasaba. **We will clarify this in the text at revision stage.**

**Question 4.  ** Consent - age or other criteria for child assent is not stated. Also, for study withdrawal only the caregiver is considered. Children also should have the ability to decide about study withdrawal. **Response 4.  ** The age of child assent in the trial is 8 years. Assent is sought from any potential participant aged 8 or more. Written assent is also sought at follow-up from minors who reach the age of 8 years during the course of the study.   Finally, if a child of 8 or more refuses to assent to participant in the study (whether or not a parent has given informed consent) this will be enough for the child to be excluded from the study. These are all clearly laid out in the manual of operations. **We will add these clarifications to the paper at revision stage.** **Question** **5. ** Quality of life assessment is mentioned as an overarching aim. Quality of life is a very important consideration for youth with sickle cell disease. Surprisingly, quality of life is relegated to “other outcomes” which diminished its importance in this trial. I’d encourage the investigators to reconsider this. Further, two different generic quality of life assessments are mentioned: EQ 5D Y and PedsQL generic core scale. Which scale will be used in analyses.  Will EQ SD Y be used for the cost effectiveness substudy and PedsQL for other analyses?  The protocol should rectify this.  Finally, I think the investigators miss an important opportunity to examine disease specific quality of life of children with sickle cell disease and would encourage them to add the PedsQL Sickle Cell module to their battery of instruments. **Response 5.  ** We completely agree with the reviewer that quality of life is a very important outcome, however, H-PRIME is a light touch pragmatic trial with multiple follow-ups being managed by a limited study team. Our aim is to keep the CRFs as simple as possible. We confirm that PedsQL will be used for QoL analysis and that ED-5D will be used for health economics. **We will add text to clarify this at revision stage.** The PedsQL sickle module is quite involving, has not been validated in Africa, and comes at additional financial cost that are beyond our funding envelope. At this stage in the trial, therefore, we are afraid that we are not able to change the protocol to include the use of this additional instrument. **We will add text to highlight these issues as potential limitations of the trial at revision stage.** **Question** **6. ** Health economics sub study – the perspective to inform the proposed cost effectiveness analysis is at the healthcare provider level.  Far more important, in my opinion, is consideration of a societal perspective.  The investigators do consider time lost from school but should also consider caregiver costs such as time lost to work for child illness, medical appointments, etc.

**Response 6.  ** We appreciate the reviewer’s arguments; however, the funding envelope for the trial was not sufficient to add a health economic analysis from the societal perspective. We will perform these analyses once the trial is over and we know impacts on cost-related issues such as transfusions and hospital admission. **We will note these limitations and additions in the Discussion section at revision stage**. **Question** **7. ** Clinical assessment #8 it is unclear how cardiac arrhythmias will be identified – via ECG monitoring or reliance on presence of symptoms only. **Response 7.  ** We state that these will include “suspected or proven cardiac arrhythmia”. Suspected arrhythmias will be based on clinical history while proven arrhythmia will be based on clinical observation of the cardiac rhythm.  We have an ECG machine at one of the sites but not all. Where possible we will confirm with ECG but that will not be the defining basis for the diagnosis. **We will elaborate on this point under our description of assessment 8 at revision stage.**

# Response

**Published:** 2026-07-09

**Authors:** Thomas N. Williams

**Affiliations:** KEMRI, Kenya

**Q1. **Power analysis: The assumptions guiding the power analyses for each hypothesis is unclear - are these guided by the literature (if so, provide citations) or fully investigator driven.  The assumption for 5% loss to follow up over a 4-year period of time seems to be unrealistic and could compromise study results.

**R1. **Our assumption of 5% loss to follow up over 4 years is in line with levels that we’ve experienced in a range of other trials within the region. For example, in the ARROW trial, a five-year follow-up trial conducted in patients with HIV in Uganda and Zimbabwe ( https://pubmed.ncbi.nlm.nih.gov/23473847/), loss to follow-up was only 2.7% and loss to follow-up in the REACH trial has remained at <5% after 10 years. We’ve added these statements to the section on sample size. Regarding our power calculations for mortality, we state on page 8 that “Data from a range of recent relevant trials (18,29) suggest that a range of mortality rates is plausible among children treated with low-dose hydroxyurea in Africa”. The references to two papers by Tshilolo and Abdullahi are the basis for the figures displayed in Table 1. Regarding the rates of hospital admission for malaria, the pre-intervention rate was derived from Macharia et al, 2017, while the figure for all-cause hospital admission, was based on data from Uyoga et al, 2019. Both studies were conducted in an area with lower malaria endemicity than Uganda and therefore probably underestimate the true power of the trial for these events. We have added these references to the power calculation section of our paper and clarified these assumptions.

**Q2. **Randomization - size of permuted blocks for randomization needs clarification. Further, while it is clearly stated that multiple children in a family would be assigned the same treatment, how this affects permuted block size and the overall randomization scheme is unclear and needs to be addressed.

**R2. **Two different sizes of permuted blocks were used but we are not making these sizes public at this stage for reasons of allocation concealment. ‘Family randomization’ was treated as a separate stratum within the weight band stratification so there was one randomization for each family. We have clarified these points in our revised paper.

**Q3. **Language of study materials available to participants including consent, assent and scales such as quality of life scales is not stated and needs to be.

**R3. **All these materials are available in both English and all 5 local languages - Ateso, Kumam, Lugwere, Luganda, and Lumasaba. We have added this in our revised paper.

**Q4.  **Consent - age or other criteria for child assent is not stated. Also, for study withdrawal only the caregiver is considered. Children also should have the ability to decide about study withdrawal.

**R4. **The age of child assent in the trial is 8 years. Assent is sought from any potential participant aged 8 or more. Written assent is also sought at follow-up from minors who reach the age of 8 years during the course of the study.   Finally, if a child of 8 or more refuses to assent to participant in the study (whether or not a parent has given informed consent) this will be enough for the child to be excluded from the study. These are all clearly laid out in the manual of operations. We have added these points in our revised paper.

**Q** **5. **Quality of life assessment is mentioned as an overarching aim. Quality of life is a very important consideration for youth with sickle cell disease. Surprisingly, quality of life is relegated to “other outcomes” which diminished its importance in this trial. I’d encourage the investigators to reconsider this. Further, two different generic quality of life assessments are mentioned: EQ 5D Y and PedsQL generic core scale. Which scale will be used in analyses.  Will EQ SD Y be used for the cost effectiveness substudy and PedsQL for other analyses?  The protocol should rectify this.  Finally, I think the investigators miss an important opportunity to examine disease specific quality of life of children with sickle cell disease and would encourage them to add the PedsQL Sickle Cell module to their battery of instruments.

**R5. **We completely agree with the reviewer that quality of life is a very important outcome, however, H-PRIME is a light touch pragmatic trial with multiple follow-ups being managed by a limited study team. Our aim is to keep the CRFs as simple as possible. We confirm that PedsQL will be used for QoL analysis and that ED-5D will be used for health economics. We have added text to clarify this. The PedsQL sickle module is quite involving, has not been validated in Africa, and comes at additional financial cost that are beyond our funding envelope. At this stage in the trial, therefore, we are afraid that we are not able to change the protocol to include the use of this additional instrument. We have added text and limitations these points in our revised paper.

**Q** **6. **Health economics sub study – the perspective to inform the proposed cost effectiveness analysis is at the healthcare provider level.  Far more important, in my opinion, is consideration of a societal perspective.  The investigators do consider time lost from school but should also consider caregiver costs such as time lost to work for child illness, medical appointments, etc.

**R6. **We appreciate the reviewer’s arguments; however, the funding envelope for the trial was not sufficient to add a health economic analysis from the societal perspective. We will perform these analyses once the trial is over and we know impacts on cost-related issues such as transfusions and hospital admission. We have noted these limitations and additions in the Discussion section.

**Q** **7. **Clinical assessment #8 it is unclear how cardiac arrhythmias will be identified – via ECG monitoring or reliance on presence of symptoms only.

**R7. **We state that these will include “suspected or proven cardiac arrhythmia”. Suspected arrhythmias will be based on clinical history while proven arrhythmia will be based on clinical observation of the cardiac rhythm.  We have an ECG machine at one of the sites but not all. Where possible we will confirm with ECG but that will not be the defining basis for the diagnosis. We have elaborated on this point in the relevant part of the section on clinical assessment.
